# Monitoring α-synuclein ubiquitination dynamics reveals key endosomal effectors mediating its trafficking and degradation

**DOI:** 10.1126/sciadv.add8910

**Published:** 2023-06-14

**Authors:** Dmitry Zenko, Jade Marsh, Andrew R. Castle, Rahel Lewin, Roman Fischer, George K. Tofaris

**Affiliations:** ^1^ Nuffield Department of Clinical Neurosciences, University of Oxford, Oxford, UK.; ^2^ Kavli Institute for Nanoscience Discovery, University of Oxford, Oxford, UK.; ^3^ Target Discovery Institute, Nuffield Department of Medicine, University of Oxford, Oxford, UK.

## Abstract

While defective α-synuclein homeostasis is central to Parkinson’s pathogenesis, fundamental questions about its degradation remain unresolved. We have developed a bimolecular fluorescence complementation assay in living cells to monitor de novo ubiquitination of α-synuclein and identified lysine residues 45, 58, and 60 as critical ubiquitination sites for its degradation. This is mediated by NBR1 binding and entry into endosomes in a process that involves ESCRT I-III for subsequent lysosomal degradation. Autophagy or the autophagic chaperone Hsc70 is dispensable for this pathway. Antibodies against diglycine-modified α-synuclein peptides confirmed that endogenous α-synuclein is similarly ubiquitinated in the brain and targeted to lysosomes in primary and iPSC-derived neurons. Ubiquitinated α-synuclein was detected in Lewy bodies and cellular models of aggregation, suggesting that it may be entrapped with endo/lysosomes in inclusions. Our data elucidate the intracellular trafficking of de novo ubiquitinated α-synuclein and provide tools for investigating the rapidly turned-over fraction of this disease-causing protein.

## Teaser

De novo ubiquitinated α-synuclein is targeted to endosomes via NBR1 and ESCRT I-III for lysosomal degradation.

## INTRODUCTION

α-Synuclein (*SNCA*) gene multiplications increase intraneuronal α-synuclein levels causing early-onset familial Parkinson’s disease (PD) (*1*), and defective α-synuclein turnover may contribute to α-synuclein accumulation in sporadic PD (*2*). Most cellular proteins are selectively targeted for degradation by conjugation to a ubiquitin chain. This modification involves activation of ubiquitin by the ubiquitin-activating enzyme (E1), transfer of the reactive ubiquitin to a ubiquitin-conjugating enzyme (E2), and then conjugation by a ubiquitin ligase (E3) to a protein substrate or a preceding ubiquitin to form a ubiquitin chain. Ubiquitin contains seven lysine residues, each of which can be linked to the C terminus of another ubiquitin molecule through an isopeptide bond. While formation of ubiquitin chains in which ubiquitin molecules are covalently linked through their K48 or K11 residues leads to the degradation of cytosolic proteins by 26*S* proteasomes, attachment of chains linked through K63 residues to membrane-associated proteins targets them for lysosomal degradation. Although ubiquitinated α-synuclein has been detected in the normal and disease brain (*3*, *4*), it remains unclear how this modification regulates α-synuclein turnover under physiological conditions, as both proteasomes and lysosomes (*5*–*7*), as well as multiple E3 ligases (*8*–*10*), have been implicated in its degradation. Currently, there is no consensus on how ubiquitin signaling regulates the turnover of physiological α-synuclein with some studies also suggesting that ubiquitination occurs after α-synuclein aggregation (*11*). It is also unknown whether α-synuclein ubiquitination in cell lines that was reported in previous studies reflects how this modification occurs in cultured neurons or brain. In this study, we have developed and validated tools to visualize the ubiquitination of α-synuclein de novo in living cells. We have mapped its intracellular trafficking to endosomes for lysosomal degradation and identified Neighbor of BRCA1 gene 1 (NBR1) and Endosomal Sorting Complexes Required for Transport (ESCRT) I-III as key effectors in a pathway that does not require autophagy.

## RESULTS

### Bimolecular fluorescence complementation enables the visualization of α-synuclein ubiquitination in living cells

**Fig. 1.**
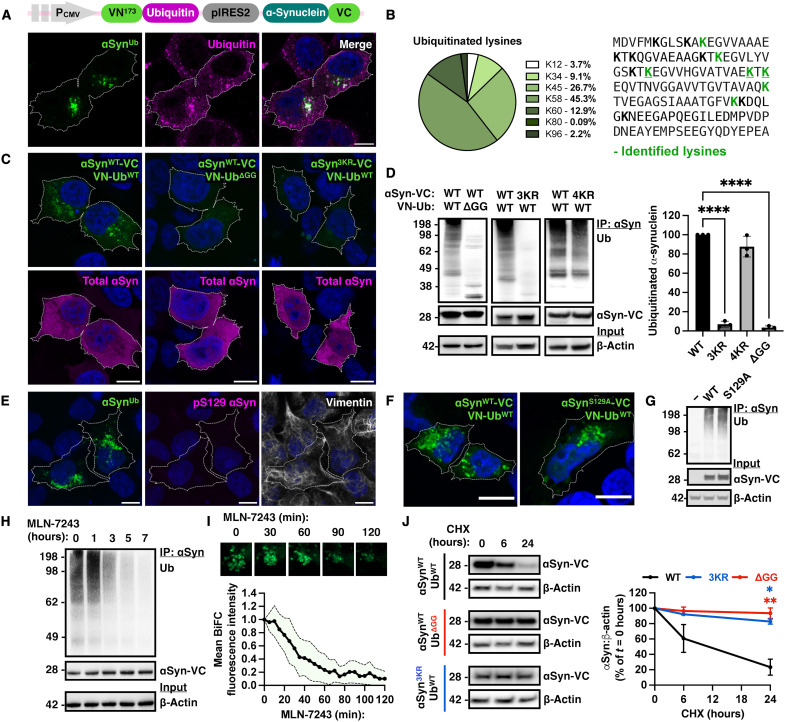
BiFC monitors α-synuclein ubiquitination. (**A**) Construct used for bicistronic expression of VN–ubiquitin (Ub) and α-synuclein (αSyn)–VC (BiFC, green) in HEK293 cells, exhibiting complementation that localizes in ubiquitin-positive intracellular foci. (**B**) α-Synuclein sequence windows of GG-modified peptides identified by mass spectrometry and peak intensities, shown as percentage to the corresponding unmodified peptides after immunoprecipitation with anti–α-synuclein antibodies. (**C**) Ubiquitin^ΔGG^ (middle) and triple mutant α-synuclein^3KR^ (right) that cannot form isopeptide bonds abolished complementation. Note similar expression of all constructs as stained with Syn1 antibody that recognizes total α-synuclein. (**D**) Immunoprecipitation (IP) of the WT, 3KR, or 4KR mutant α-synuclein with Syn1 followed by immunoblotting with anti-ubiquitin antibodies confirmed that α-synuclein is ubiquitinated in this model at specific lysine residues as detected by mass spectrometry. (**E**) BiFC expression used throughout the study does not lead to pathological phosphorylation of α-synuclein at S129 or colocalization with vimentin. S129A mutation on α-synuclein does not prevent (**F**) the formation of fluorescent foci or (**G**) detection of polyubiquitination following Syn1 immunoprecipitation and immunoblotting for ubiquitin. Addition of the E1 inhibitor MLN-7243 followed by (**H**) α-synuclein immunoprecipitation and immunoblotting for ubiquitin or (**I**) time-lapse microscopy in living cells (also see movie S1), demonstrating that ubiquitination is fast and dynamic (*n* = 30 cells from *n* = 3 independent experiments). (**J**) Cycloheximide (CHX) chase experiments followed by immunoblotting showed degradation of WT α-synuclein–VC when coexpressed with WT VN-ubiquitin. In contrast, WT α-synuclein–VC coexpressed with ΔGG VN-ubiquitin or 3KR mutant α-synuclein–VC coexpressed with WT VN-ubiquitin was not degraded. Data are representative of *n* = 3 independent experiments and shown as means ± SEM. *P < 0.05, **P < 0.01, and ****P < 0.0001 by one-way (D) or two-way (J) ANOVA followed by Tukey’s multiple comparison test. Scale bars, 10 μm.

Tracking the dynamics of ubiquitinated α-synuclein has not been possible because tools that visualize and monitor its degradation by different pathways have been lacking. This is mainly due to the diffuse localization of α-synuclein in cultured cells, including neurons. We tested whether bimolecular fluorescence complementation (BiFC) can be used to monitor the trafficking or degradation of the rapidly turned-over fraction of α-synuclein in living cells. We fused ubiquitin to the N-terminal (VN173) fragment (VN-ubiquitin) and α-synuclein to the C-terminal (VC155) fragment (α-synuclein–VC) of the Venus fluorescent protein. Both fragments were cloned into the pIRES2 bicistronic vector to drive the simultaneous expression of both fusion proteins under the control of the same promoter (Fig. 1A and fig. S1A). We observed that complementation in human embryonic kidney (HEK) 293 or SH-SY5Y cells led to the formation of fluorescent puncta (Fig. 1A and fig. S1B), suggesting that ubiquitinated α-synuclein localizes to specific foci such as organelles or inclusion bodies. We confirmed that the foci were immunoreactive for ubiquitin (Fig. 1A and fig. S1B). Immunoprecipitation of α-synuclein and immunoblotting with anti-ubiquitin antibodies showed that the fluorescent signal in cells was due to polyubiquitination of α-synuclein only when both constructs were expressed (fig. S1, C and D). We also compared the distribution of untagged α-synuclein to α-synuclein–VC expressed alone in HEK293 cells by fractionation or in SH-SY5Y cells knockout for endogenous α-synuclein by immunofluorescence. These experiments showed that fusion of the VC fragment to the C terminus of α-synuclein per se does not change its localization (fig. S1, E and F). To identify the lysine residues that become ubiquitinated, we performed mass spectrometry on immunoprecipitated α-synuclein and identified diglycine (GG)–modified α-synuclein peptides in lysates from cells expressing wild-type (WT) α-synuclein–VC and VN-ubiquitin. The most abundant GG-modified lysine residues of α-synuclein were at positions K45, K58, and K60 (Fig. 1B). To confirm that the identified lysine residues are the predominant sites of α-synuclein ubiquitination, we used site-directed mutagenesis to substitute these lysine residues to arginine. We also assessed the specificity of the interaction by generating mutant ubiquitin that lacks the C-terminal GG motif at positions 95 and 96 (ΔGG) that is required for conjugation to a lysine residue on a protein substrate. Under both conditions, the BiFC signal was completely abolished in cells (Fig. 1C). Immunoprecipitation from lysates expressing α-synuclein–VC and VN-ubiquitin^ΔGG^ or triple K45R/K58R/K60R mutant α-synuclein–VC (herein referred to as 3KR) with WT VN-ubiquitin showed that ubiquitinated α-synuclein was no longer detectable with anti-ubiquitin antibodies (Fig. 1D). Using the Syn1 antibody, which recognizes total α-synuclein, we confirmed that the constructs were expressed under all three conditions by immunofluorescence (Fig. 1C) and immunoblotting (Fig. 1D). In a separate construct, we mutated the previously reported additional ubiquitination sites at K12, K21, K23, and K96 for comparison (*9*, *12*). Expression of K12R/K21R/K23R/K96R (4KR) mutant α-synuclein–VC with WT VN-ubiquitin did not prevent the ubiquitination as revealed by immunoblotting (Fig. 1D). Collectively, these experiments indicate that BiFC foci in our cellular assay correspond to a specific pool of α-synuclein that undergoes ubiquitination primarily but not exclusively at K45, K58, and K60. Lastly, we tested and confirmed that at the low level of expression used for these experiments, α-synuclein was not phosphorylated at Serine 129 (pS129), which is a common pathological modification of aggregated α-synuclein, and did not colocalize with vimentin, a marker of aggresomes as evident by immunofluorescence (Fig. 1E). pS129 α-synuclein was also tested by immunoblotting following fractionation; pathological phosphorylation and aggregation were only seen when the construct was expressed at high levels as expected for this aggregation-prone protein (fig. S1, G and H). In addition, mutation of S129 to alanine (S129A) did not prevent the formation of these foci (Fig. 1F) or the detection of a polyubiquitinated smear after Syn1 immunoprecipitation and anti-ubiquitin immunoblotting (Fig. 1G), suggesting that α-synuclein ubiquitination occurs independently of this modification.

We then treated HEK293 cells expressing BiFC with a nontoxic dose of MLN-7243 (500 nM), a small-molecule inhibitor of the ubiquitin-activating enzyme Uba1. We monitored α-synuclein ubiquitination by immunoblotting (Fig. 1H) and more accurately by time-lapse microscopy in living cells (Fig. 1I and movie S1). These experiments showed that ubiquitinated α-synuclein decreased in a time-dependent manner and was almost completely abolished within 120 min when assessed in living cells (Fig. 1I). These data show that complementation in the context of ubiquitination is fast, dynamic, and responsive to pharmacological treatment. To assess whether ubiquitination triggers degradation, we performed cycloheximide chase experiments and compared the degradation kinetics of WT and 3KR α-synuclein–VC when coexpressed in HEK293 cells with WT VN-ubiquitin. Bicistronic expression of WT α-synuclein–VC with a nonconjugatable form of VN-ubiquitin (ΔGG) was also included as an additional control. Cells were treated with cycloheximide (10 μg/ml) to inhibit protein synthesis, and levels of α-synuclein were monitored by immunoblotting at the indicated time points (Fig. 1J). These experiments showed that the levels of WT α-synuclein–VC, which reflect the monomeric pool of α-synuclein that is visualized when ubiquitinated in our assay, were reduced after 6 hours of treatment to 62.7 ± 23.5% and after 24 hours to 23.2 ± 6.1% compared to baseline (*t* = 0), indicating that ubiquitinated α-synuclein is degraded (Fig. 1J). In contrast, α-synuclein levels remained unchanged when the 3KR α-synuclein–VC was expressed with WT ubiquitin (98 ± 3.6%) or when WT α-synuclein–VC was expressed with VN-ubiquitin^ΔGG^ (90.3 ± 5.9%). Thus, de novo ubiquitination of α-synuclein in cells at one or more of the three identified lysine residues (K45, K58, and K60) is a signal for its degradation.

### De novo ubiquitination targets α-synuclein to the lysosome, not the proteasome

**Fig. 2.**
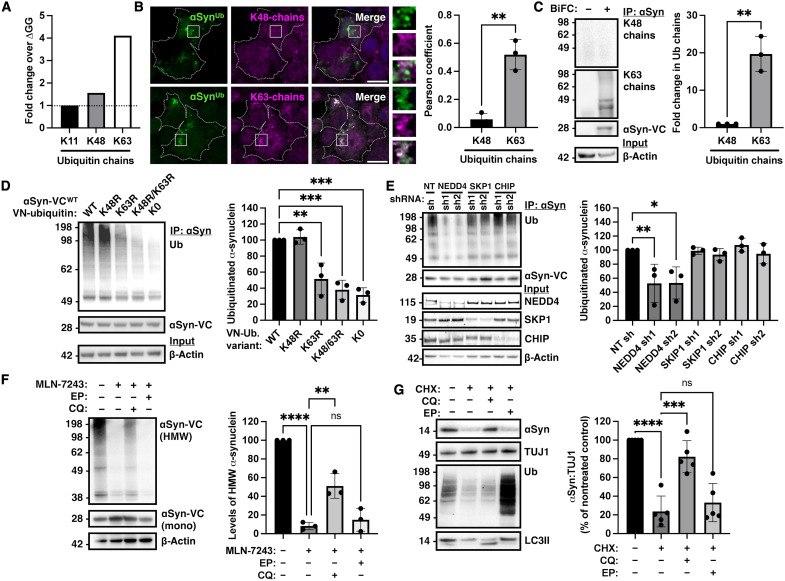
De novo ubiquitination of α-synuclein is a signal for its lysosomal rather than proteasomal degradation. (**A**) Mass spectrometry following immunoprecipitation of ubiquitinated α-synuclein identified predominantly K63-linked chains and, to a lesser extent, K48- and K11-linked chains. (**B**) Foci of ubiquitinated α-synuclein were immunoreactive with anti-K63–linked ubiquitin antibodies (bottom) but not anti-K48–linked ubiquitin antibodies (top), as quantified with Pearson’ s coefficient. (**C**) Immunoprecipitation of α-synuclein followed by immunoblotting with either K63-linked specific or K48-linked anti-ubiquitin antibodies. (**D**) Expression of K63R mutant but not K48R mutant VN-ubiquitin reduced the ubiquitination of α-synuclein. Ubiquitinated α-synuclein was reduced when K63R/K48R or K0 ubiquitin was expressed. (**E**) Using two shRNA per target each in three independent experiments, knockdown of NEDD4 but not other E3 ligases reduced the ubiquitination of α-synuclein. (**F**) Cells expressing α-synuclein–VC and VN-ubiquitin were lysed at baseline, after treatment with MLN-7243 (500 nM) for 6 hours alone or in combination with either chloroquine (CQ; 50 μM) or epoxomicin (EP; 500 nM). Ubiquitinated α-synuclein was stabilized only by lysosomal inhibition (*n* = 3). (**G**) Primary neurons were treated with cycloheximide alone or together with epoxomicin (500 nM) or chloroquine (50 μM), showing that α-synuclein is degraded by the lysosome (*n* = 5). The effect of inhibitors was confirmed by LC3 and ubiquitin immunoblotting. Data are shown as means ± SEM. **P < 0.01, ***P < 0.001, and ****P < 0.0001 by two-sided unpaired Student’s *t* test (B and C) or one-way analysis of variance (ANOVA) (D to G) followed by Tukey’s multiple comparison test. ns, not significant. Scale bars, 10 μm.

Our mass spectrometry analysis showed that K63-linked ubiquitin chains that are involved in endosomal trafficking were fourfold more abundant when compared to K48- or K11-linked chains in α-synuclein immunoprecipitates from WT BiFC-expressing HEK293 cell lysates compared to ΔGG ubiquitin/WT α-synuclein controls (Fig. 2A). To confirm that α-synuclein per se is conjugated to K63-linked chains in this system, we used two complementary approaches. First, we used chain-specific antibodies and showed that both BiFC-positive foci (Fig. 2B) and immunoprecipitated α-synuclein (Fig. 2C) were immunoreactive with anti-K63–linked but not anti-K48–linked ubiquitin chain antibodies. Second, we generated K48R, K63R, or K48R/K63R mutant ubiquitin that cannot form K48, K63, or both K48/K63 polyubiquitin chains, respectively. We found that the K48R VN-ubiquitin mutation did not impair α-synuclein ubiquitination, whereas the K63R mutant VN-ubiquitin reduced the ubiquitination of α-synuclein (Fig. 2D). Expression of the double ubiquitin mutant K48R/K63R did not reduce α-synuclein ubiquitination more than what was observed with the single K63R mutant. In addition, expression of the K0 VN-ubiquitin mutant that does not contain any lysine residues also reduced the signal intensity, suggesting that ubiquitin chains rather than multiple monoubiquitin conjugates form on α-synuclein in this system. We also assessed the function of endogenous E3 ligases that were previously implicated in α-synuclein ubiquitination. To this end, we used two short hairpin–mediated RNA (shRNA) per target to knock down neural precursor cell expressed developmentally down-regulated protein 4 (NEDD4) (*9*), S-phase kinase-associated protein 1 (SKP1) (*10*), or C terminus of heat shock cognate (Hsc)70-interacting protein (CHIP) (*13*), followed by transient transfection with BiFC in HEK293 cells. Ubiquitination was assessed by α-synuclein immunoprecipitation and immunoblotting with anti-ubiquitin antibodies. These experiments showed that knockdown of endogenous NEDD4, an E3 ligase that forms K63-linked ubiquitin chains, reduced the ubiquitination of α-synuclein in this system (Fig. 2E). Knockdown efficiency of the E3s was confirmed for each shRNA by immunoblotting as shown in Fig. 2E and quantified in fig. S2. In agreement with these results, we found that following treatment with the E1 inhibitor MLN-7243 (500 nM), ubiquitinated α-synuclein as assessed by immunoblotting was stabilized by chloroquine (50 μM) that neutralizes the acidic pH within lysosomes but not epoxomicin (500 nM) that inhibits the proteasome (Fig. 2F). To assess the relevance of these findings in the neuronal context, we revisited the mode of degradation of endogenous α-synuclein in primary cortical neurons at day in vitro 21 (DIV21) following treatment with a nontoxic dose of cycloheximide (10 μg/ml for 24 hours). These experiments confirmed the prediction of our BiFC assay in cell lines, demonstrating that under conditions that do not involve protein overexpression, neuronal degradation of endogenous α-synuclein was prevented by lysosomal but not proteasomal inhibition (Fig. 2G). In control experiments, we confirmed the effects of these inhibitors by showing accumulation of polyubiquitinated substrates with epoxomicin and LC3II with chloroquine treatment (Fig. 2G). Thus, under nonaggregation conditions, our assay successfully visualizes the ubiquitinated fraction of α-synuclein, demonstrating that this fraction is targeted for degradation by the lysosome and not the proteasome.

### Ubiquitination of α-synuclein is required for its recruitment to endosomes

**Fig. 3.**
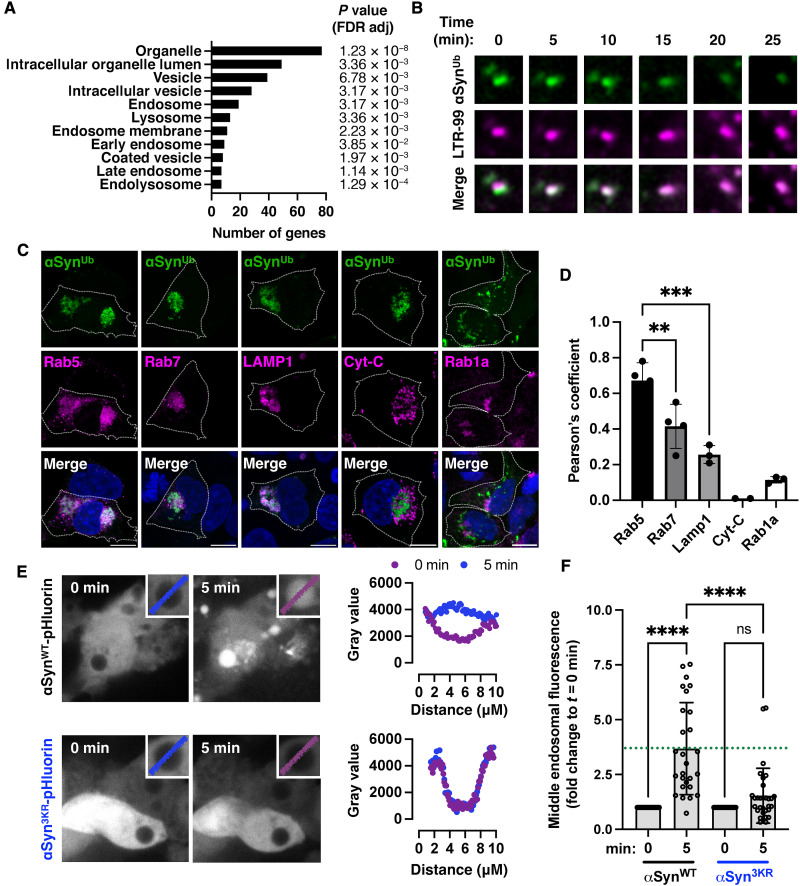
α-Synuclein ubiquitination triggers its trafficking to the endosome. (**A**) Immunoprecipitated WT α-synuclein–VC bicistronically expressed with either WT VN-ubiquitin or ΔGG VN-ubiquitin was subjected to mass spectrometry. GO enrichment analysis for proteins that were differentially associated with ubiquitinated α-synuclein identified terms in the endosomal-lysosomal pathway (table S1). (**B**) Live imaging confirmed that green fluorescence foci corresponding to ubiquitinated α-synuclein (also see movie S2) fused with LysoTracker (LTR-99) positive lysosomes, leading to loss of green signal due to degradation or quenching in the acidic pH. (**C**) Ubiquitinated α-synuclein colocalized with Rab5-positive (early) and Rab7-positive (late) endosomes or Lamp1-positive lysosomes but not mitochondria [cytochrome C (Cyt-C)–positive] or the ER-Golgi network (Rab1a). Scale bars, 10 μm. (**D**) Quantification of colocalization with Pearson’s coefficient in fixed cells. (**E**) Expression of Rab5^Q79L^ that enlarges endosomes was used to monitor the localization of α-synuclein–pHluorin (seGFP) at the endosomal membrane and its internalization, which is visualized by the intraluminal green fluorescence following neutralization of the acidic pH with chloroquine. Representative images with additional examples are shown in movies S3 and S4. (**F**) Quantification is based on 30 cells from *n* = 3 independent experiments. Images in (B), (C), and (E) are representative of *n* = 3 independent experiments, and graphs are shown as means ± SEM. **P < 0.01, ***P < 0.001, and ****P < 0.0001 by one-way ANOVA followed by Tukey’s multiple comparison test.

To determine the pathway by which ubiquitinated α-synuclein is trafficked inside cells, we performed mass spectrometry to identify interacting protein complexes with α-synuclein when coexpressed with either WT or ΔGG ubiquitin. We used gene ontology (GO) term analysis to define enriched functions or components within identified proteins. Enrichment scores, the degree to which a list of proteins in a GO term is represented within the protein list when compared to the total list of proteins tested, were plotted for GO terms that were significant (*P* value threshold of 10^−3^). This analysis revealed terms enriched in intracellular organelles, membrane-enclosed lumen, endosomes, and lysosomes (Fig. 3A and table S1). We therefore asked whether the fluorescent foci of ubiquitinated α-synuclein colocalize with intracellular organelles. Time-lapse confocal live cell microscopy showed fusion of BiFC foci with LysoTracker Red DND-99–positive organelles corresponding to late endosomes or lysosomes (Fig. 3B and movie S2). We further assessed these trafficking events in fixed cells where we found that the BiFC signal colocalized with Rab5 (early endosome marker), Rab7 (late endosome marker), and Lamp1 (lysosomal marker) but not cytochrome C (mitochondrial marker) or Rab1a [endoplasmic reticulum (ER) to Golgi trafficking effector also involved in macroautophagy] as shown in Fig. 3C and quantified in Fig. 3D. This analysis showed that the extent of colocalization of the BiFC signal was lower with Rab7- versus Rab5-positive or Lamp1- versus Rab5-positive organelles, suggesting that recruitment starts at the early endosomes. We detected minimal colocalization between BiFC foci and the 20*S* proteasome or VPS13C (fig. S3).

Ubiquitination of endosomal substrates promotes their transfer from the limiting membrane of the endosome to the lumen in a process termed involution. To determine whether ubiquitination triggers the involution of α-synuclein at early endosomes, we coexpressed Rab5^Q79L^, a guanosine triphosphatase–deficient Rab5 that is known to enlarge early endosomes, together with WT or 3KR mutant α-synuclein C-terminally tagged with a pH-sensitive super ecliptic green fluorescent protein (GFP) (pHluorin). Under these conditions, only WT α-synuclein that can be ubiquitinated but not the 3KR mutant accumulated inside the endosomal lumen when their acidic pH was quenched acutely with 500 μM chloroquine, as shown in Fig. 3E and movies S3 and S4 and quantified in Fig. 3F. Collectively, these data establish that de novo ubiquitination of nonaggregated α-synuclein in cells is a signal for its trafficking to endosomes.

### α-Synuclein recruitment and entry into endosomes involves the autophagic adaptor NBR1 and ESCRT I-III

**Fig. 4.**
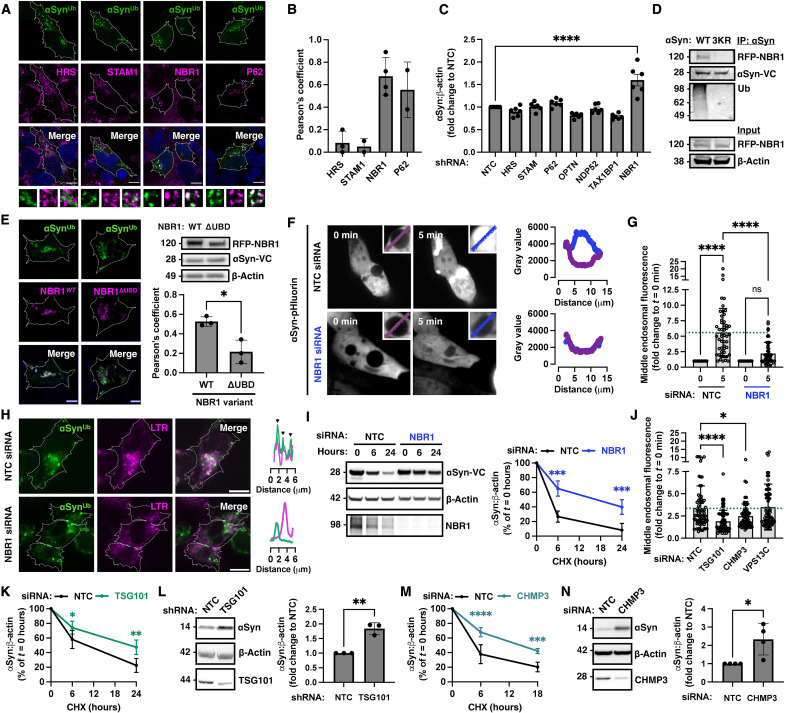
Ubiquitinated α-synuclein is degraded by lysosomes in an NBR1-dependent manner involving ESCRT I-III. (**A**) Colocalization of green foci of ubiquitinated α-synuclein with NBR1 and p62 but not ESCRT-0 adaptors as quantified in (**B**) by Pearson’s coefficient (*n* = 2 to 3, 50 cells per replicate). (**C**) Knockdown of NBR1 but not other ubiquitin-binding adaptors (two shRNA per target, *n* = 3 each) increased endogenous α-synuclein levels in SH-SY5Y cells at steady state. (**D**) RFP-NBR1 coimmunoprecipitated with ubiquitinated α-synuclein when coexpressed with WT but not 3KR mutant α-synuclein–VC. (**E**) Deletion of the ubiquitin-binding domain (UBD) from NBR1 reduced its colocalization with ubiquitinated α-synuclein (*n* = 3 independent experiments, 50 cells per replicate). Immunoblot shows equal level of expression of WT or ΔUBD NBR1 in cells. (**F**) NBR1 knockdown prevents the internalization of α-synuclein into Rab5^Q79L^-induced enlarged endosomes as monitored with α-synuclein–pHluorin and quantified in (**G**). Also see movies S5 and S6. (**H**) NBR1 knockdown prevents the fusion of BiFC foci with lysosomes as also shown in movies S7 and S8. (**I**) Cycloheximide chase after NBR1 knockdown in BiFC-expressing HEK293 cells revealed reduced ubiquitin-mediated α-synuclein degradation (*n* = 3). (**J**) Knockdown of either TSG101 or CHMP3 but not VPS13C reduced the internalization of α-synuclein into Rab5^Q79L^-induced enlarged endosomes as monitored with α-synuclein–pHluorin and quantified in the graph. Also see movies S9 to S11. (**K**) TSG101 knockdown reduced the rate of ubiquitin-mediated α-synuclein degradation in BiFC HEK 293 cells (*n* = 3) and (**L**) increased endogenous α-synuclein levels in SH-SY5Y cells (*n* = 3). (**M**) CHMP3 knockdown reduced the rate of ubiquitin-mediated α-synuclein degradation in BiFC HEK293 cells (*n* = 5) and (**N**) increased endogenous α-synuclein levels in SH-SY5Y cells (*n* = 4). Data are shown as means ± SEM. *P < 0.05, **P < 0.01, and ****P < 0.0001 by two-sided unpaired Student’s *t* test (E, L, and N), one-way (C, G, and J), or two-way (I, K, and M) ANOVA followed by Tukey’s multiple comparison test. Scale bars, 10 μm.

Unlike integral membrane proteins that are typically sorted by the ESCRT complex, α-synuclein is loosely associated with membranes and present in the cytosol (*14*). We therefore sought to identify the relevant adaptor that recruits ubiquitinated α-synuclein to endosomes. We initially asked whether the fluorescent puncta colocalized with autophagic or ESCRT-0 adaptors that contain a ubiquitin binding domain. Pearson’s coefficient analysis did not show any colocalization with Signal transducing adaptor molecule (STAM) or Hepatocyte growth factor-regulated tyrosine kinase substrate (HRS) (ESCRT-0 components), as shown in Fig. 4A and quantified in Fig. 4B. Instead, ubiquitinated α-synuclein strongly colocalized with the autophagy adaptors NBR1 and p62 (Fig. 4, A and B). To functionally assess the effects of ubiquitin binding adaptors, we knocked down NBR1, STAM, HRS, p62, Optineurin (OPTN), Nuclear domain 10 protein 52 (NDP52), and Tax1 binding protein 1 (TAX1BP1) using two shRNA per target in SH-SY5Y cells that endogenously express α-synuclein. We found that knockdown of NBR1 increased intracellular levels of α-synuclein, whereas depletion of the other adaptors had no effect (Fig. 4C and fig. S4). We focused on NBR1 as it was the only adaptor that both colocalized with BiFC in HEK293 cells and increased endogenous α-synuclein levels at steady state when knocked down in SH-SY5Y cells. Initially, we tested whether ubiquitination is necessary for this interaction, using two complementary approaches. First, we tested and confirmed that WT but not 3KR mutant α-synuclein coimmunoprecipitated with red fluorescent protein (RFP)–NBR1 (Fig. 4D). Second, we expressed a truncated form of RFP-NBR1 at residue 906 lacking the ubiquitin binding domain (NBR1^ΔUBD^) and showed that this deletion reduced the colocalization of NBR1 with fluorescent foci of ubiquitinated α-synuclein (Fig. 4E). We then visualized the entry of α-synuclein into Rab5^Q79L^-dilated endosomes using time-lapse confocal microscopy. Using WT α-synuclein–pHluorin to monitor entry into endosomes, we found that NBR1 knockdown markedly reduced the detection of fluorescent signal inside the endosomal lumen when compared to nontargeting control (NTC) small interfering RNA (siRNA) after acute quenching of the endosomal pH with 500 μM chloroquine shown in Fig. 4F and movies S5 and S6 and quantified in Fig. 4G. In accordance with these findings, we also showed that knockdown of NBR1 prevented the subsequent fusion of BiFC foci with lysosomes (Fig. 4H and movie S7 compared to movie S8). Lastly, we found that siRNA knockdown of NBR1 reduced the rate of clearance of α-synuclein–VC in cycloheximide-chase experiments in HEK293 cells expressing WT BiFC (Fig. 4I).

To further delineate the mechanism of involution, we investigated the function of late ESCRT complexes that are involved in membrane scission (*15*) because we noticed that VPS4, which regulates the disassembly of ESCRT III, was identified in our proteomic analysis (table S1). Loss of VPS4 function is associated with substantial toxicity (*15*), so, instead, we knocked down tumor susceptibility gene 101 (Tsg101, an ESCRT-I component) or charged multivesicular body protein 3 (CHMP3) also known as Vacuolar protein sorting-associated protein 24 (VPS24, an ESCRT-III component). In addition, we knocked down VPS13C that is a PD-associated gene (*16*), also identified in our proteomic analysis, and implicated in endosomal membrane lipid homeostasis (*17*). Using the WT α-synuclein–pHluorin assay described above to monitor entry into endosomes, we found that knockdown of either Tsg101 or CHMP3 but not VPS13C reduced the involution of α-synuclein as shown in Fig. 4J and movies S9 to S11. Successful knockdown of these effectors was confirmed by immunoblotting as shown in Fig. 4 (L and N) and fig. S4 with relevant quantifications in fig. S4. We next tested whether Tsg101 or CHMP3 regulate α-synuclein levels using two approaches: First, we found that Tsg101 or CHMP3 knockdown reduced the degradation of α-synuclein–VC in HEK293 cells following cycloheximide treatment (Fig. 4, K and M, and fig. S4C for representative immunoblots), and, second, we showed that knockdown of Tsg101 (Fig. 4L) or CHMP3 (Fig. 4N) in SH-SY5Y cells increased α-synuclein levels at steady state. Collectively, using genetic and pharmacological approaches, our data show that following ubiquitination, α-synuclein involution into endosomes depends on NBR1 and ESCRT I-III, which is required for subsequent fusion with and degradation by the lysosome.

### Autophagy is dispensable for the degradation of ubiquitinated α-synuclein

**Fig. 5.**
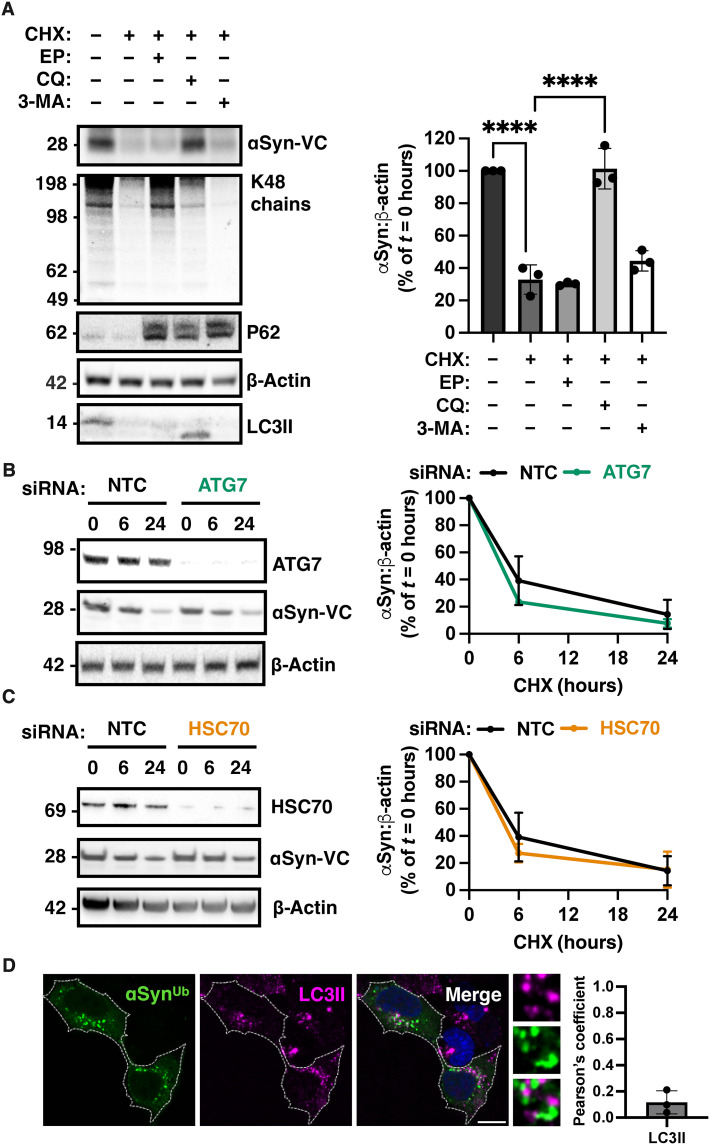
Autophagy is dispensable for the degradation of ubiquitinated α-synuclein. (**A**) Bicistronic expression of α-synuclein–VC and VN-ubiquitin and degradation kinetics of α-synuclein–VC as measured by immunoblotting at baseline or after addition of cycloheximide and nontoxic concentrations of epoxomicin (500 nM), chloroquine (50 μM), or 3-MA (10 mM) for 24 hours. The effect of inhibitors was confirmed by LC3II, p62, and ubiquitin immunoblotting. The immunoblot is a representative of *n* = 3 independent experiments, and quantification is shown in the graph. (**B**) siRNA-mediated knockdown of ATG7 did not modify the rate of clearance of α-synuclein–VC following cycloheximide chase as illustrated by the representative immunoblot and quantified in the accompanied graph (*n* = 3 independent experiments). (**C**) siRNA-mediated knockdown of Hsc70 did not modify the rate of clearance of α-synuclein–VC following BiFC expression and cycloheximide chase as illustrated by the representative immunoblot and quantified in the accompanying graph (*n* = 3 independent experiments). (**D**) No colocalization of green foci of ubiquitinated α-synuclein with LC3II after chloroquine treatment was seen by immunofluorescence confocal microscopy as quantified in the graph. Data are shown as means ± SEM. ****P < 0.0001 by one-way (A) ANOVA followed by Tukey’s multiple comparison test. Scale bar, 10 μm.

Endosomes fuse either directly with lysosomes or with autophagosomes forming amphisomes that subsequently fuse with lysosomes. Previous studies in human cell lines implicated both pathways in the degradation of α-synuclein when measuring total levels (*9*, *18*, *19*). To distinguish the relative contribution of these two routes and assess the relevance of macroautophagy in the degradation of the ubiquitinated fraction of α-synuclein, we performed cycloheximide-chase experiments. In these experiments, HEK293 cells were treated with cycloheximide alone or together with nontoxic doses of inhibitors of the different pathways. We found that the reduction in the levels of WT α-synuclein–VC when coexpressed with WT VN-ubiquitin was blocked by the lysosomotropic agent chloroquine (50 μM) but not the proteasomal inhibitor epoxomicin (500 nM) or 3-methyladenine (3-MA, 10 mM), a compound that blocks the formation of autophagosomes (Fig. 5A). In control experiments, we confirmed the effects of these inhibitors as follows: K48-linked ubiquitinated substrates accumulated with epoxomicin, p62 that is a substrate of autophagy and the proteasome accumulated with all inhibitors, and LC3II accumulated with chloroquine and was reduced by 3-MA as expected (*20*).

To further investigate the role of autophagic pathways, we expressed our BiFC construct in cells where autophagy related 7 (ATG7), an E1-like enzyme required for autophagy, was knocked down. We found that following cycloheximide treatment, the rate of clearance of monomeric α-synuclein–VC, which we showed earlier to be the fraction that is ubiquitinated in this assay, was unaffected (Fig. 5B). We also found that knockdown Hsc70, a key effector of chaperone-mediated autophagy and microautophagy, is dispensable for the degradation of α-synuclein–VC (Fig. 5C). Therefore, neither macroautophagy nor chaperone-mediated autophagy is essential for the degradation of ubiquitinated α-synuclein–VC. In agreement with this finding, fluorescent BiFC puncta did not colocalize with LC3II after treatment with chloroquine (Fig. 5D).

### α-Synuclein is ubiquitinated in the brain and targeted to lysosomes in primary neurons

**Fig. 6.**
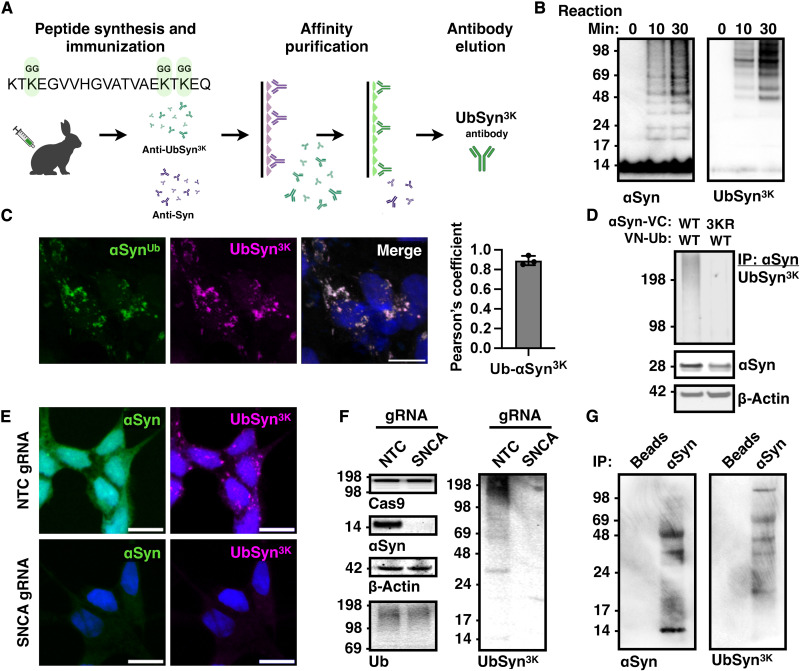
Generation of antibodies against ubiquitinated α-synuclein. (**A**) Polyclonal antibodies raised against GG-modified α-synuclein peptide (anti-UbSyn^3K^) were generated and purified as shown. (**B**) Anti-UbSyn^3K^ antibodies recognized principally ubiquitinated recombinant α-synuclein generated by recombinant NEDD4 in vitro. (**C**) Anti-UbSyn^3K^ antibodies immunostained BiFC-positive foci in HEK293 cells and (**D**) recognized a smear after Syn1 immunoprecipitation from cells expressing WT but not 3KR α-synuclein–VC with WT VN-ubiquitin. (**E**) Puncta resembling the BiFC foci were detected endogenously in WT but not α-synuclein knockout SH-SY5Y cells. (**F**) Endogenous ubiquitinated α-synuclein was detected by immunoblotting in WT but not α-synuclein knockout SH-SY5Y cells (**G**) Anti-UbSyn^3K^ antibodies recognized ubiquitinated α-synuclein (smear) but not monomer following immunoprecipitation with Syn1 from rat brain homogenate. Scale bars, 10 μm.

To assess whether the dynamics of α-synuclein ubiquitination and its interactome that we delineated using the BiFC assay are relevant to the physiological trafficking of α-synuclein in neuronal tissues, we generated antibodies against an α-synuclein peptide conjugated to GG dipeptides at K45, K58, and K60 (KTK^45GG^EGVVHGVATVAEK^58GG^TK^60GG^EQ), termed anti-UbSyn^3K^ (Fig. 6A). First, we tested in a purified system whether this antibody recognizes human recombinant α-synuclein that is ubiquitinated by the E3 ligase NEDD4. We previously showed by mass spectrometry that NEDD4 ubiquitinates α-synuclein at K21, K45, K58, and K96 (*9*). These experiments confirmed that anti-UbSyn^3K^ antibodies recognize predominantly ubiquitinated α-synuclein and not the monomeric protein at 14 kDa (Fig. 6B). We also confirmed that the anti-UbSyn^3K^ antibody recognizes by immunofluorescence the BiFC-positive puncta (Fig. 6C) and by immunoblotting a smear (polyubiquitinated α-synuclein) after Syn1 immunoprecipitation of WT but not 3KR mutant BiFC when expressed in HEK293 cells (Fig. 6D). To investigate the specificity of anti-UbSyn^3K^ antibodies for endogenous α-synuclein, we knocked out *SNCA* in SH-SY5Y dopamine-like cells using CRISPR-Cas9 and compared the staining pattern and immunoblotting to WT cells. These experiments showed a specific punctate pattern by immunofluorescence with anti-UbSyn^3K^ in WT but not *SNCA* knockout cells, suggesting that endogenously ubiquitinated α-synuclein localizes in a similar way as we detected with our BiFC assay (Fig. 6E). In addition, immunoblotting of cell lysates with anti-UbSyn^3K^ antibodies identified a smear corresponding to ubiquitinated α-synuclein that was not detected in *SNCA* knockout cells (Fig. 6F). Anti-UbSyn^3K^ antibodies recognized ubiquitinated α-synuclein from adult rat brain homogenate that was immunoprecipitated with Syn1 antibodies (Fig. 6G). Thus, endogenous brain α-synuclein is ubiquitinated in vivo on the same lysine residues (K45, K58, and K60) as identified by our BiFC assay in culture, suggesting that ubiquitination serves a similar role in endosomal trafficking in the brain.

**Fig. 7.**
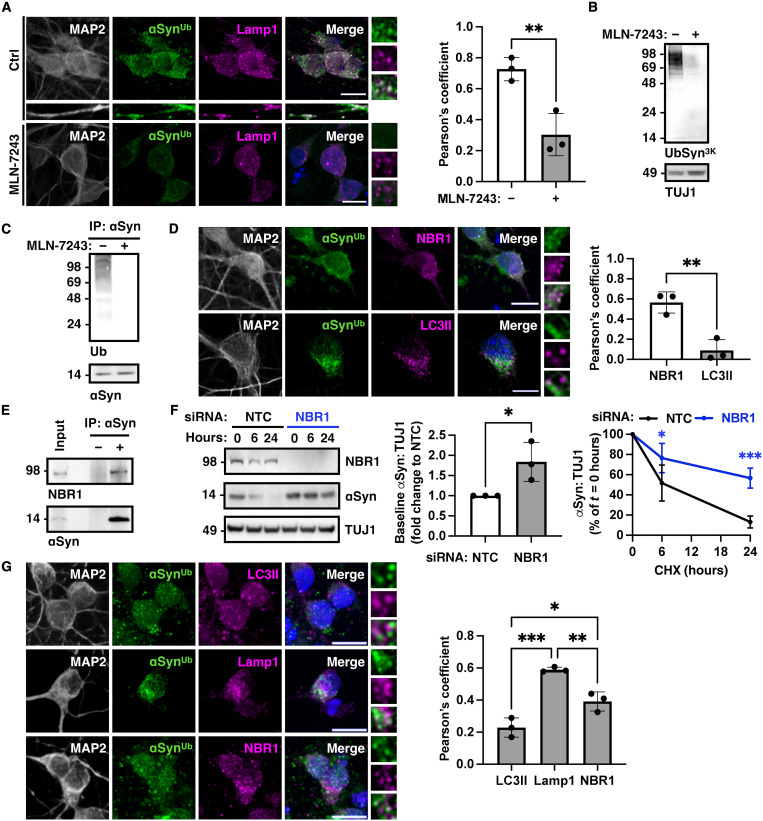
Neuronal α-synuclein is ubiquitinated and targeted to lysosomes in an NBR1-dependent manner. (**A**) Anti-UbSyn^3K^ antibodies stained foci of endogenous neuronal α-synuclein that colocalized with Lamp1, and this colocalization was lost following E1 inhibition with MLN-7243 as quantified with Pearson’s coefficient. (**B**) Immunoblotting of neuronal lysates with anti-UbSyn^3K^ identified a smear that was not detectable following treatment with MLN-7243. (**C**) Immunoprecipitation of α-synuclein with Syn1 and immunoblotting for ubiquitin at baseline or after MLN-7243 treatment confirmed the presence of ubiquitinated α-synuclein in rat cortical neurons at DIV21. (**D**) Colocalization of anti-UbSyn^3K^–positive foci with NBR1 at baseline but not LC3II-positive autophagosomes after treatment with chloroquine. (**E**) Coimmunoprecipitation of endogenous NBR1 with α-synuclein from rat brain lysate. (**F**) siRNA knockdown of NBR1 in primary rat cortical neurons increased basal levels (*t* = 0) of neuronal α-synuclein as quantified in the histogram and reduced the rate of its degradation. (**G**) Anti-UbSyn^3K^–positive foci colocalized with NBR1 and Lamp1 at baseline and only minimally with LC3II-positive autophagosomes in human iPSC-derived dopaminergic neurons. Data are representative of *n* = 3 independent experiments and shown as means ± SEM. *P < 0.05, **P < 0.01, and ***P < 0.001, by two-sided unpaired Student’s *t* test (A, D, and F, baseline), two-way (F, time course) or one-way (G) ANOVA followed by Tukey’s multiple comparison test. Scale bars, 10 μm.

To further investigate this prediction, we used primary rat cortical neurons that were cultured in vitro for 21 days. Staining with anti-Lamp1 and anti-UbSyn^3K^ antibodies showed that endogenous ubiquitinated α-synuclein colocalized with Lamp1-positive organelles both in the neuronal cell bodies and processes at baseline, and this colocalization was reduced in neurons treated with the E1 inhibitor MLN-7243 (Fig. 7A). Under these conditions, immunoblotting of neuronal lysates with anti-UbSyn^3K^ antibodies revealed a smear, which was not seen after MLN-7243 treatment (Fig. 7B), indicating that UbSyn^3K^ antibodies detect ubiquitinated α-synuclein in primary neurons. We further confirmed by Syn1 immunoprecipitation (total α-synuclein) and anti-ubiquitin immunoblotting the presence of ubiquitinated α-synuclein at baseline but not after MLN-7243 treatment (Fig. 7C and quantified in fig. S5A). Similar to our observations with BiFC expression in HEK293 cells, anti-UbSyn^3K^–positive puncta colocalized with endogenous NBR1 in primary neurons at baseline (Fig. 7D) but minimally with LC3II-positive autophagosomes that accumulated upon chloroquine treatment (Fig. 7D). α-Synuclein coimmunoprecipitated with endogenous NBR1 from rat brain lysate indicating an interaction between the two proteins (Fig. 7E). siRNA-mediated NBR1 knockdown in primary neurons increased the basal levels of α-synuclein by approximately 1.8-fold (levels at time zero) and reduced its rate of degradation following treatment with cycloheximide as shown in Fig. 7F. These data suggest that in neurons, ubiquitination of α-synuclein is a signal that targets the protein to the lysosome in an NBR1-dependent manner as we found with BiFC in human cell lines. We further confirmed that in human induced pluripotent stem cell (iPSC)-derived dopaminergic neurons (characterized in fig. S5, B and C), anti-UbSyn^3K^–positive puncta colocalized principally with Lamp1-positive organelles and NBR1 and only minimally with LC3II-positive autophagosomes (Fig. 7G).

### Ubiquitinated α-synuclein is detected in pathological inclusions

**Fig. 8.**
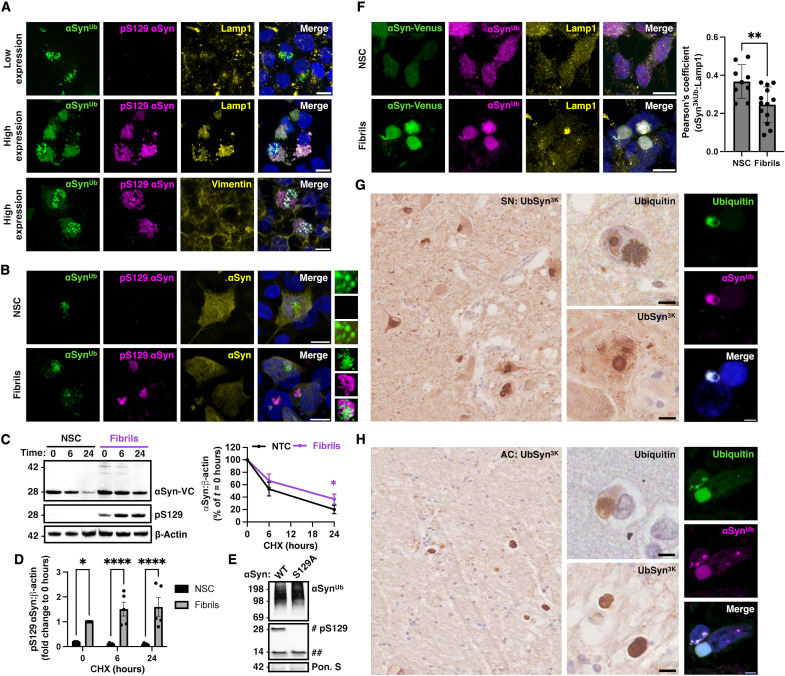
Ubiquitinated α-synuclein is detected in pathological aggregates. (**A**) High but not low expression of BiFC led to the formation of pS129-positive aggregates with entrapped Lamp1-positive lysosomes. Aggregates under conditions of high BiFC expression were vimentin-negative. (**B**) Addition of α-synuclein fibrils (1 μM) to low BiFC-expressing HEK293 cells led to the clustering of BiFC foci with pS129 α-synuclein–positive aggregates. (**C**) The rate of clearance of α-synuclein–VC as assessed by cycloheximide chase was reduced and (**D**) pS129 modified α-synuclein increased after seeding with fibrils compared to NSC as illustrated by the representative immunoblots and quantified in the accompanying graphs (*n* = 5 independent experiments). (**E**) Expression of S129A mutant α-synuclein did not prevent ubiquitination after seeded aggregation as assessed by anti-UbSyn^3K^ immunoblotting (#α-synuclein–VC; ##endogenous α-synuclein). (**F**) Formation of seeded aggregates in α-synuclein–Venus expressing cells led to redistribution of anti-UbSyn^3K^ staining from the cytosol to the inclusions and reduced colocalization with Lamp1-positive organelles in the cytosol as quantified with Pearson’s coefficient; *n* = 3 independent experiments, each dot representing the image average of >50 cells. Immunohistochemistry of human sections with anti-UbSyn^3K^ antibodies revealed punctate staining and immunoreactive Lewy bodies in (**G**) the SN and (**H**) the AC that resembled and colocalized with ubiquitin staining. Images are representative examples from a total of *n* = 3 PD cases. *P < 0.05, **P < 0.01, and ****P < 0.0001 by two-way ANOVA (C) followed by Tukey’s multiple comparison test or two-sided unpaired Student’s *t* test (D and F). Scale bars, 10 μm.

We showed earlier that high expression of the BiFC construct induces the formation of pathological α-synuclein pS129 (fig. S1, G and H). We exploited these conditions to investigate the fate of 3K ubiquitinated α-synuclein when the protein aggregates. We observed clustering of BiFC-positive puncta with Lamp1 and Lamp1-positive organelles within pS129-positive aggregates (Fig. 8A). In contrast, we did not observe colocalization with vimentin (Fig. 8A), a marker of aggresomes that form via a distinct process (*21*). We assessed this finding in the context of seeded aggregation with the addition of recombinant α-synuclein fibrils (fig. S6, A to D). We found that seeding of HEK293 cells expressing low levels of the BiFC construct also induced pS129-positive aggregates that colocalized with BiFC-positive vesicles (Fig. 8B). In cells with seeded aggregates, the degradation of α-synuclein–VC as assessed by immunoblotting of total cell lysates was reduced (Fig. 8C), pS129 α-synuclein–VC accumulated (Fig. 8D), and total α-synuclein–VC levels were increased at each time point (fig. S6E). For the chase experiments, fibrils were added 24 hours after BiFC expression, and cells were treated with cycloheximide 24 hours after fibril addition. Mutation of S129A did not modify ubiquitination after seeded aggregation as assessed by anti-UbSyn^3K^ immunoblotting (Fig. 8E). Lastly, we used fibrils to induce seeding in HEK293 cells stably expressing α-synuclein–Venus and monitored α-synuclein ubiquitination at 48 hours using anti-UbSyn^3K^ antibodies. Immunocytochemistry with anti-UbSyn^3K^ revealed a change in the pattern of ubiquitinated α-synuclein from punctate staining in the cytosol of nonseeded cells (NSC) to localization in Venus-positive aggregates following treatment with fibrils (Fig. 8F). Under these pathological conditions, UbSyn^3K^ colocalized with Lamp1-positive organelles within the inclusions as shown in Fig. 8F and by orthogonal images in fig. S6F, whereas UbSyn^3K^ colocalization with Lamp1-positive organelles in the cytosol was reduced as quantified in the graph in Fig. 8F. In control experiments, we confirmed that total Lamp1 levels were unchanged (fig. S6G). To investigate the relevance of this finding to the human condition, we asked whether postmortem brain sections [substantia nigra (SN) and anterior cingulate cortex (AC)] from patients with PD (*n* = 3 cases studied) are stained with anti-UbSyn^3K^ antibodies. Anti-UbSyn^3K^ antibodies revealed a punctate background staining and immunostaining of the pathological inclusions termed Lewy bodies (Fig. 8, G and H). Lewy bodies stained with anti-UbSyn^3K^ antibodies resembled the staining pattern seen with anti-ubiquitin antibodies that typically recognize the ring of the inclusions in the SN (Fig. 8G) and the whole inclusion in the cortex (Fig. 8H). We also confirmed the colocalization of anti-UbSyn^3K^ and anti-ubiquitin staining with double labeling immunofluorescence confocal microscopy (Fig. 8, G and H). In control experiments, we tested and confirmed that anti-UbSyn^3K^ antibodies do not detect monomeric α-synuclein by immunoblot in total human brain lysates from control or PD brains (fig. S7A). In addition, immunoblotting with anti-UbSyn^3K^ antibodies following Syn1 immunoprecipitation from human brain lysate revealed a smear that was also detected with anti-ubiquitin antibodies (fig. S7B) but no monomer. These data suggest that, in the human brain, UbSyn^3K^ is entrapped into aggregates under pathological conditions.

## DISCUSSION

Despite numerous studies on α-synuclein degradation, its regulation by the ubiquitin system has not been fully resolved, and the neuronal relevance of studies performed in cell lines has never been assessed. Using diverse approaches in living or fixed cells, including two cell lines (HEK293 and SH-SY5Y), rat cortical neurons and human iPSC-derived dopaminergic neurons, our study establishes that de novo ubiquitination triggers the trafficking of α-synuclein to endosomes for lysosomal degradation. These experiments were performed under nonaggregation conditions and further validated in primary neurons where α-synuclein is found in the cytosol or loosely associated with membranes (*14*, *22*). In cortical neurons or iPSC-derived dopaminergic neurons, we observed colocalization of endogenously ubiquitinated α-synuclein with Lamp1-positive organelles, which, in neurons, signify the spectrum of endolysosomes as they are transported to cell bodies from presynaptic terminals in a process associated with increased acidification and degradative capacity (*23*). Although we have confirmed the reversibility of the resulting complementation signal and relevance to endogenous proteins, we cannot exclude the possibility that the interaction of the two Venus fragments may enhance a particular pattern of ubiquitination. Therefore, it remains to be seen whether ubiquitination at other lysine residues have any physiological relevance for α-synuclein trafficking, degradation, or aggregation.

We showed that K63-linked ubiquitin conjugation on K45, K58, or K60 of α-synuclein is required for this trafficking step. K63-linked ubiquitination mediates the trafficking of transmembrane proteins such as receptors to endosomes by ESCRT-0 (*24*) or cytosolic aggregates and organelles to autophagy via NBR1, p62, and other autophagy adaptors (*25*). In our experiments, ubiquitinated α-synuclein minimally colocalized with LC3II in human cells or primary neurons, and previous studies showed that brain-specific conditional ATG7 knockout in mice did not cause α-synuclein accumulation in ubiquitinated inclusions (*26*). Thus, autophagy is dispensable for the lysosomal degradation of the ubiquitinated fraction of α-synuclein. This finding is also consistent with our current and previous studies that identified the E3 ligase NEDD4 (*9*), which forms K63-linked chains on α-synuclein (positions K45 and K58), as well as ubiquitin specific peptidase 8 (USP8) (*27*) as key regulators of α-synuclein degradation. No K48-linked ubiquitin chains formed on α-synuclein de novo at baseline or after inhibition of the proteasome and cycloheximide-chase experiments showed that endogenous α-synuclein is not degraded by the proteasome in primary neurons in agreement with earlier findings (*28*). It is noteworthy that in some of the earlier studies, proteasome inhibitors were applied over prolonged incubations (>24 hours), which exert broader effects on cellular homeostasis (*28*, *29*) and therefore may indirectly modify α-synuclein turnover. Under certain conditions, overexpressed α-synuclein and potentially other natively unfolded proteins may be degraded by the proteasome in a ubiquitin-independent manner (*6*, *30*), and studies in vitro or in vivo have not detected accumulation of ubiquitinated α-synuclein following proteasomal inhibition (*31*). Our data do not exclude the possibility that proteasomes degrade a small fraction of α-synuclein in neuronal subcompartments in vivo or under certain conditions that favor formation of microaggregates such as aging or overexpression (*31*). This withstanding, de novo ubiquitination of α-synuclein under nonaggregation conditions is primarily a signal that triggers endosomal localization for degradation by lysosomes and not the proteasome.

Ubiquitination of the identified lysine residues 45, 58, or 60 maps to the KTKEGV core repeat motifs 4 and 5 within the proposed amphipathic helix 3 and 4 of α-synuclein (*32*), which mediates membrane binding. Engineering of additional lysine residues in this region (E35K + E46K + E61K within repeat motifs 3, 4, and 5) were shown to enhance the interaction with membranes (*33*). It is therefore conceivable that modification of K45, K58, and K60 by ubiquitination may serve as a checkpoint to isolate from the membrane potentially damaged α-synuclein and/or preferentially target the cytosolic monomeric form or the fraction that is coming off the membrane.

Another important finding from our work is the identification of NBR1 as a critical adaptor for the involution of ubiquitinated α-synuclein into endosomes and its turnover in human cells and primary neurons. The yeast orthologue Nbr1 was previously shown to mediate the trafficking of cytosolic proteins to the vacuole (*34*), but NBR1 has only been shown to mediate autophagy in mammalian cells (*35*, *36*). We found that NBR1 is required for the internalization of α-synuclein into endosomes. Accordingly, knockdown of NBR1 stalled the trafficking of ubiquitinated α-synuclein along the endosomal-lysosomal pathway. Thus, our study suggests a previously unidentified mode of processing of ubiquitinated proteins at endosomal membranes via NBR1 in a process that does not require LC3. The mechanism described herein has some similarities with “endosomal microautophagy” or “microautophagy” where Hsc70 directs proteins to the late endosome where membrane invagination via ESCRT I-III delivers cytosolic proteins to lysosomes (*37*). Our data indicate that ESCRT I-III regulate the delivery of α-synuclein into endosomes. However, unlike microautophagy, we found that Hsc70 is dispensable and ubiquitination is necessary for the recruitment of α-synuclein to endosomes. We also showed that ubiquitinated α-synuclein localized at early and late endosomal compartments. Whether this fundamental mechanism is specific to α-synuclein or applicable to other cytosolic proteins remains to be seen.

In the context of disease mechanisms, our data offer a potential explanation linking α-synuclein accumulation with genome-wide association studies, which implicate the endosomal and lysosomal pathways in PD pathogenesis (*2*). The relevance of endosomes in disease is also supported by the finding that endosome-derived exosomes isolated from patients with PD exhibit a twofold increase in α-synuclein content (*38*, *39*). NBR1 was shown to colocalize with α-synuclein in Lewy bodies in PD brains, but not with other aggregated proteins in neurodegenerative diseases (*40*). We previously showed that Lewy bodies are immunostained with antibodies against K63-linked but not K48-linked ubiquitin chains (*27*).

Ubiquitination of soluble α-synuclein at positions K45, K58, and K60 that mediates endosomal trafficking is distinct from K12 and K21 ubiquitination that were identified on α-synuclein fibrils isolated from human brain extract (*12*, *41*). This is expected because unlike the conformation of monomeric, nonmembrane-bound α-synuclein (*42*), positions K45, K58, and K60 are located in the inner core of α-synuclein fibrils (*41*) and not exposed to the cytosol for modification. Therefore, the previously reported ubiquitination of N-terminal lysine residues on fibrils may occur after assembly (*43*) or may denote a different proteolytic mechanism. In this context, the most parsimonious explanation for the staining of pathological inclusions with anti-UbSyn^3K^ antibodies in our cellular models of aggregation and Lewy bodies is that K45-, K58-, and K60-ubiquitinated α-synuclein is entrapped with endosomes/lysosomes into aggregates, offering one potential mechanism by which trafficking is disrupted in disease. Such ubiquitinated species would be removed during sarkosyl-based extraction that is used for the isolation and characterization of fibrillar assemblies. Previous electron microscopy studies of intact Lewy bodies showed that they are composed of dysmorphic organelles including endosomes and lysosomes that are interspersed with fibrils (*44*, *45*). It is therefore conceivable that at the ultrastructural level, 3K ubiquitinated α-synuclein in Lewy bodies is associated with these trapped endosomes. Sequestration of relevant effectors such as NBR1 (*40*) or endosomes-lysosomes (*44*, *45*) in inclusions may impair efficient degradation of α-synuclein as shown in our and other (*46*) models of seeded aggregation. Endosomes are also key compartments for the internalization of exogenous α-synuclein assemblies that may contribute to the spread of pathology and disease progression (*47*). Therefore, endosomes could be the focus of intraneuronal convergence of physiological and pathological α-synuclein promoting further aggregation, especially when the rate of transport to or degradation by the lysosome is impaired.

α-Synuclein is thought to be mainly a long-lived protein, which has made it difficult until now to study its rapidly degraded pool. Our study demonstrates that BiFC is a valuable tool in the study of dynamic changes in response to ubiquitination of α-synuclein and potentially other proteins and faithfully recapitulates its endogenous ubiquitination in the brain and neurons. It is therefore likely that these assays can also be used to accelerate the discovery of novel therapeutics targeting α-synuclein homeostasis.

## MATERIALS AND METHODS

### Cell culture and transfection

HEK293 [American Type Culture Collection (ATCC) CRL-1573] cells were cultured in Dulbecco’s modified Eagle medium (DMEM) supplemented with 10% heat-inactivated fetal bovine serum (FBS) and 1× nonessential amino acids, 1× GlutaMAX, and 1× penicillin-streptomycin (Pen-Strep; Gibco). Human neuroblastoma SH-SY5Y cells (ATCC CRL-2266) were cultured in DMEM/F12 (1:1 mix) supplemented with 10% FBS, 1× nonessential amino acids, 1× GlutaMAX, and 1× Pen-Strep. Cells were routinely passaged by lifting in TrypLE (Thermo Fisher Scientific). All transfections were carried out using Lipofectamine 2000 according to the manufacturer’s instructions, unless otherwise stated. Cells were transfected in six-well plates and then replated onto poly-l-lysine–coated glass coverslips. Transfection of siRNA was achieved using the DharmaFECT 1 transfection reagent according to the manufacturer’s instructions. The siRNA sequences used for this study are summarized in table S2.

### Dissection and cortical neurons culture

Cerebral cortices were isolated from the brains of Sprague-Dawley rat embryos (E16, Envigo, UK). Isolated cortices were mechanically trypsinized in the 0.25% TrypLE solution (Gibco) for 15 min at 37°C, followed by mechanical dissociation in ice-cold Hank’s balanced salt solution (Invitrogen, UK) supplemented with 10 mM Hepes (pH 7.4). Cell suspension was then filtered through a 40-μm sterile polyethersulfone (PES) filter and was plated onto poly-d-lysine–coated (Sigma-Aldrich) and laminin-coated (Sigma-Aldrich) coverslips at a density of 8 × 10^4^ cells per well or six-well plate at a density of 1 × 10^6^ cells per well. Cortical neurons were maintained in presence of Neurobasal Plus medium (Gibco) supplemented with B27 Plus solution (Gibco), 0.5 mM GlutaMAX, and 0.5% Pen-Strep (Gibco) under an atmospheric condition of 5% CO_2_ and 95% air at 37°C. To eliminate contaminating neural progenitor cells, CultureOne Supplement (Thermo Fisher Scientific, UK) was added to a complete medium for the first 2 days in culture. Neuronal cultures for maintained for approximately 10 to 14 days to achieve full neuronal maturity prior to all experiments.

### Generation of iPSC-derived midbrain dopaminergic neurons

Dopaminergic neurons were derived from a healthy control iPSC line as previously described (*48*). iPSCs were seeded onto Geltrex-coated six-well plates, expanded until >80% confluency and mTeSR1 medium was changed to DIV0 medium (2 μM A83-01 and 100 nM LDN in neural induction base medium). DIV1 to DIV4 medium contains 2 μM A83-01, 100 nM LDN, SHH C25II (300 ng/ml), 2 μM purmorphamine, and fibroblast growth factor 8a (FGF8a; 200 ng/ml) in neural induction base medium. Three micromolars of CHIR-99021 was added from day 3 until day 12. DIV5 to DIV6 medium contains 100 nM LDN, SHH C25II (300 ng/ml), 2 μM purmorphamine, 3 μM CHIR-99021, and FGF8a (200 ng/ml) in neural induction base medium. DIV7 to DIV10 medium contains 100 nM LDN and 3 μM CHIR-99021 in neural induction base medium. DIV11 to DIV19 medium contains brain-derived neurotrophic factor (20 ng/ml), glial cell line–derived neurotrophic factor (20 ng/ml), transforming growth factor–β3 (1 ng/ml), 10 μM DAPT, 200 μM ascorbic acid, 500 μM db–adenosine 3′,5′-monophosphate in neural differentiation medium, and laminin (1 μg/ml) is added from 17 to 25 days of differentiation. At DIV20, cells were replated into Geltrex-coated plates or coverslips. From DIV21, medium changes were done with DIV11 to DIV19 components until analysis time points were reached.

### Generation of lentiviral particles and shRNA transduction

The shRNA sequences used for this study are summarized in table S3. All viruses were produced using packaging plasmid psPAX.2, envelope plasmid pMD2.G, shRNA plasmid, and a polyethylenimine (PEI)–based transfection protocol. Briefly, 70 to 90% confluent HEK 293 T cells were transfected, followed by medium replacement and medium collection. Conditioned media were spun at 400*g* for 5 min, and the supernatant was filtered (0.22-μm PES), aliquoted, and stored at −80°C. For gene knockdown studies, conditioned media containing lentiviral particles were added to SH-SY5Y cells for 48 hours. Cells were then selected using puromycin (2 μg/ml) for 72 hours before being processed for the Western blot analysis as described above and immunoblotting. Protein knockdown was tested using commercially available antibodies.

#### CRISPR-Cas9 knockout in cells

Single-guide RNA (sgRNA) for the α-synuclein gene (*SNCA*) (GCTGCTGAGAAAACCAAACA) or nontarget control (GGATACCTGGGCCGACTTTC) was designed using the online tool (http://crispor.tefor.net/) and cloned into the Bbs I site of the pNGx_LV_g003 lentivirus plasmid. To generate virus, HEK293T cells were transfected with the sgRNA transfer plasmid and psPAX.2 and pMD2.G plasmids using the PEI method. After 12 hours, the media were replaced, and lentiviral-conditioned media were harvested 48 hours later, clarified by centrifugation at 400*g* for 10 min, and filtered through a 0.22-μm PES membrane. The conditioned media were aliquoted and stored at −80°C. SH-SY5Y cells stably expressing Cas9 cells were generated by transfection of the Lenti-Cas9-2A-Blast plasmid using Lipofectamine 2000 and selected using blasticidin (2.5 μg/ml). Clonal SH-SY5Y cells stably expressing Cas9 were transduced with 200 μl of conditioned lentiviral medium and selected 48 hours later with medium containing puromycin (2 μg/ml) for 3 days before used for experiments. α-Synuclein knockout was confirmed by immunoblotting using commercially available antibodies.

### Immunoblotting

Denatured and reduced samples were resolved by SDS–polyacrylamide gel electrophoresis (PAGE) using NuPAGE 4 to 12% bis-tris gels and transferred to nitrocellulose using the iBlot 2 Gel Transfer system. Signals were detected either using horseradish peroxidase–conjugated goat anti-mouse or goat anti-rabbit immunoglobulin G (IgG) (Abcam), followed by enhanced chemiluminescence prime detection reagent (Amersham) or by use of Dylight 680–conjugated goat anti-mouse and Dylight 800–conjugated goat anti-rabbit fluorescent secondary antibodies. Blots were imaged on a Bio-Rad ChemiDoc, and band intensities of unsaturated immunoblots were analyzed and quantified by densitometry using ImageJ. Relevant primary and secondary antibodies are shown in tables S4 and S5.

### Biochemical detection of α-synuclein degradation

Cellular levels of α-synuclein were determined by immunoblotting using a cycloheximide chase analysis. Briefly, HEK293 cells transiently expressing applicable constructs were plated into 12-well culture plates. On the day of experiments, cells were treated with cycloheximide (10 μg/ml) for indicated time points before washing in ice-cold phosphate-buffered saline (PBS) and lysing in extraction buffer [140 mM NaCl, 10 mM tris-HCl (pH 8.0), 1 mM EDTA, 1% Triton X-100, 0.1% SDS, 50 mM *N*-ethylmaleimide (NEM), and 1 mM phenylmethylsulfonyl fluoride (PMSF)] supplemented with a standard protease inhibitor cocktail (Roche). Lysates were clarified by centrifugation and analyzed by immunoblotting as described above.

### Immunoprecipitation and detection of α-synuclein ubiquitination

Immunoprecipitations were performed in HEK293, SH-SY5Y, rat cortical neurons, or brain lysate. On the day of the experiment, cells or tissue were lysed in extraction buffer [140 mM NaCl, 10 mM tris-HCl (pH 8.0), 1 mM EDTA, 1% Triton X-100, 0.1% SDS, 50 mM NEM, and 1 mM PMSF] supplemented with a standard protease inhibitor cocktail (Roche). For detections of ubiquitination, SDS concentration was raised to 2%, and samples were incubated for 30 min at 4°C and sonicated for 20 s at 30% amplitude before being diluted in a standard extraction buffer for final concentration of SDS to reach 0.1%. Samples were clarified by centrifugation at 14,000*g* and then incubated with Syn1 antibody (BD Transduction) and Dynabeads Protein G (Thermo Fisher Scientific) overnight at 4°C. Next day, samples were washed three times in a standard extraction buffer, boiled, and resolved by SDS-PAGE as described above. For liquid chromatography–mass spectrometry, immunoprecipitation samples were digested on beads using a RapidDigest (Promega) according to the manufacturer’s instructions.

### Fractionation of cell lysates

For crude separation into cytosolic, membrane, and insoluble fractions, the cell monolayer was washed once with ice-cold PBS. Fresh PBS supplemented with protease and phosphatase inhibitor cocktails was added, and cells were scraped and lysed by sonication, followed by centrifugation at 700*g* for 5 min to remove cell debris. The supernatants were spun at maximum speed in a microcentrifuge (~24,000*g*) for 20 min to pellet the membrane fraction, which was resuspended in Pierce radioimmunoprecipitation assay (RIPA) buffer (Thermo Fisher Scientific) supplemented with protease and phosphatase inhibitor cocktails. The supernatants from the ~24,000*g* spin were spun at 100,000*g* for 1 hour to generate cytosolic (PBS-soluble) and insoluble (final pellet) fractions. The final pellet was resuspended in RIPA with the SDS concentration adjusted up from 0.1 to 2%. For separation into soluble and insoluble fractions only, cells were lysed in extraction buffer [140 mM NaCl, 10 mM tris-HCl (pH 8.0), 1 mM EDTA, 1% Triton X-100, 0.1% SDS, 50 mM NEM, and 1 mM PMSF] supplemented with protease and phosphatase inhibitor cocktails. Cell lysates were centrifuged at 10,000*g* for 20 min, and the resulting supernatant was spun at 100,000*g* for 1 hour to obtain the soluble fraction and the insoluble pellet, which was resuspended in the above extraction buffer with the SDS concentration adjusted to 2%. In either case, samples were analyzed by immunoblotting as described above.

### Mass spectrometry and data analysis

Mass spectrometry was performed as described previously (*43*). Beads were spun down and resuspended in 150 μl of trypsin buffer (Promega). Samples were reduced using 10 μl of dithiothreitol (DTT) reducing agent (final concentration, 10 mM) and incubated for 45 min at room temperature (RT). Samples were then alkylated with iodoacetamide (final concentration, 40 mM) and incubated for 45 min at RT. SMART Digest Trypsin (Thermo Fisher Scientific) was added in a 1:50 ratio with respect to total protein content, and samples were digested at 70°C for 1 hour. Samples were analyzed on a nano–liquid chromatography–tandem mass spectrometry system consisting of an Orbitrap Fusion Lumos or Q-Exavtive connected to a Dionex Ultimate 3000 (Thermo Fisher Scientific). Peptides were separated on an Easy-Spray column (50 cm) with a gradient of 2 to 35% ACN in 5% dimethyl sulfoxide in 0.1% formic acid over 60 min. MS1 spectra were acquired with a resolution of 120,000 and an AGC target of 4 × 10^5^. MS2 spectra were acquired in the linear ion trap after HCD fragmentation at 28% normalized collision energy for up to 35 ms and an AGC target of 4 × 10^3^. Selected precursors masses were excluded for 60 s. Data analysis was performed in MaxQuant (v1.6.2.3) (*49*). Peptide precursor ion abundance was used to compare the volume under the chromatographic signal of each peptide detected for a protein. Peptide signals were then summarized into ratios for each protein across the different conditions, yielding a fold change value. Searches were performed with a mass tolerance of 10 parts per million for precursors and 0.5 Da for fragments. Swissprot/Trembl FASTA file was used as database. Protein and peptide false discovery rates (FDRs) were set to 1% with Match between Runs option enabled.

### In vitro ubiquitination of recombinant α-synuclein

In vitro ubiquitination experiments were performed as described before (*9*). Each reaction (10 μl) typically contained 50 nM of human His^6^-Uba1 (E1 ligase, R&D Systems, UK), 500 nM of the human His^6^-UbcH5b (E2 ligase, R&D Systems, UK), 300 nM of the human His^6^-NEDD4 (E3 ligase, R&D Systems, UK), 5 μg of ubiquitin (Boston Biochem), and 50 nM of recombinant α-synuclein. All reactions were incubated at 30°C for the indicated time points in presence of 2 mM tris-buffered adenosine 5′-triphosphate. His^6^-tagged proteins were pulled-down with Ni–nitrilotriacetic acid Sepharose (Thermo Fisher Scientific), and ubiquitination of α-synuclein was assessed by immunoblotting.

### Plasmids and cloning

Human ubiquitin was polymerase chain reaction (PCR)–amplified from pcDNA3-HA-ubiquitin (gift from E. Yeh, Addgene plasmid #18712) (*50*) and cloned with a VN tag into pcDNA3. Subsequently, α-synuclein–VC and VN-ubiquitin were cloned into a single backbone with pIRES2 to drive simultaneous expression of both proteins with the same promoter, using the NEBuilder HiFi DNA assembly [New England Biolabs (NEB)] kit. Rab5^Q79L^ was generated by site-directed mutagenesis of mRFP-Rab5 (gift from A. Helenius, Addgene plasmid #4437) (*51*), using the QuikChange II site-directed mutagenesis kit (Agilent). mRFP-NBR1 was generated by replacing Rab5 cDNA in the mRFP-Rab5 plasmid with NBR1 cDNA amplified from pMXs-puro-mCherry-NBR1 (gift from J. Debnath, Addgene plasmid #74242) (*52*) using the NEBuilder HiFi DNA Assembly (NEB) kit. mRFP-NBR1^ΔUBD^ was generated by adapting mRFP-NBR1 using the QuikChange site-directed mutagenesis kit. WT α-synuclein–pHluorin was cloned using pCMV-lyso-pHluorin (gift from C. Rosenmund, Addgene plasmid #70113) (*53*), and 3KR was generated using the QuikChange II site-directed mutagenesis kit (Agilent). PCR-based site-directed mutagenesis was also performed to introduce mutations in VN-ubiquitin (K48R, K63R, K48R/K63R, and ΔGG) and α-synuclein–VC (3KR, 4KR, and S129A). All newly constructed plasmids were validated by sequencing (Eurofins).

### Generation of recombinant human α-synuclein monomer and fibrils

The pRK172 expression vector encoding WT human α-synuclein (a gift from M. Goedert) was transformed into the *Escherichia coli* strain BL21 DE3 (Stratagene, La Jolla, CA, USA). Transformed BL21 DE3 cells were cultured for 2.5 hours, and the expression of α-synuclein was induced by the addition of 0.44 mM isopropyl-β-d-thiogalactopyranoside. After culturing for a further 5 hours, bacteria were harvested and resuspended in a low-salt buffer containing 50 mM tris (pH 7.4), 20 mM NaCl, 1 mM EDTA (pH 8.0), 0.1 mM DTT, 0.1 mM PMSF, protease inhibitor cocktail (Roche), lysozyme (1 mg/ml), and deoxyribonuclease and ribonuclease (1:1000). The bacteria were lysed by a freeze-thaw cycle at −80°C, followed by probe sonication. The lysate was clarified by centrifugation and filtration through a 0.45-μm filter. α-Synuclein was purified by loading the lysate onto a HiTrap Q HP column and eluting using a gradient of 0- to 500-mm NaCl. The fractions containing α-synuclein were pooled, concentrated by ammonium sulfate precipitation and resuspended in low-salt buffer. The solution was loaded onto a HiLoad 16/600 Superdex 75 prep grade column (Cytiva), and fractions containing α-synuclein were dialyzed into PBS buffer using 3000 molecular weight cutoff (MWCO) dialysis casettes (Pierce) overnight and concentrated using Ultrafree-15, 3000 MWCO filter columns (Millipore Corp.) To pellet any aggregated material, the solution was centrifuged at 24,000*g* for 1 hour at 4°C. The protein concentration was measured by BCA, and samples were diluted to 5 mg/ml (350 μM) and stored at −80°C. Purification of α-synuclein monomer was validated by Western blotting. For fibril formation, α-synuclein monomer was incubated at 37°C under continuous shaking for 14 days in an Eppendorf thermomixer set at 1000 rpm. Generation of α-synuclein fibrils was confirmed by the Thioflavin T binding assay and electron microscopy.

### Transmission electron microscopy

Ten microliters of a fibril suspension (0.2 mg/ml) was applied to freshly glow discharged carbon 200-mesh copper grids for 2 min, blotted with filter paper, stained with 2% uranyl acetate for 10 s, then blotted, briefly passed over a water droplet, blotted, and air-dried. Grids were imaged in a FEI Tecnai 12 transmission electron microscope at 120 kV using a Gatan OneView complementary metal-oxide semiconductor camera.

### Seeded aggregation of WT human α-synuclein in cells

Clonal HEK293 cells stably expressing WT human α-synuclein fused to Venus on the C terminus or transiently transfected with the bicistronic BiFC construct were cultured in DMEM/F12 medium containing 1× GlutaMAX, 1× minimum essential medium NEAA, 10% FBS, and 1× Pen-Strep and incubated at 37°C in humidified air with 5% CO_2_. Cells were seeded with 1 μM sonicated α-synuclein fibrils in Opti-MEM containing Lipofectamine 2000 (Invitrogen) for 6 hours. The medium was replaced with routine culturing medium, and cells were incubated for 48 hours, washed with PBS, and fixed with 4% formaldehyde in PBS for immunofluorescence confocal microscopy or lysed in in extraction buffer [140 mM NaCl, 10 mM tris-HCl (pH 8.0), 1 mM EDTA, 1% Triton X-100, 0.1% SDS, 50 mM NEM, and 1 mM PMSF] supplemented with protease and phosphatase inhibitor cocktails.

### Generation of anti-ubiquitinated α-synuclein antibody

Two peptides were synthesized at high purity and confirmed by high-performance liquid chromatography and mass spectrometry: One peptide had GG conjugated to the side chain of lysine residues: KTK(*GG*)EGVVHGVATVAEK(GG)TK(*GG*)EQ. The cognate unmodified peptide was also generated: KTKEGVVHGVATVAEKTKEQ. Approximately 10 mg of the modified peptide was conjugated to keyhole limpet hemocyanin for immunization and bovine serum albumin (BSA) conjugation for monitoring production of specific IgG after the immunizations. The remaining peptide was used for affinity purification. For polyclonal antibody production, a 90-day immunization protocol was used to ensure that only immunoglobulin of the IgG isotype was produced in the antiserum. Two rabbits were immunized using three doses of the modified peptide at weeks 2, 4, and 7. The production of antibodies was monitored by dot blot analysis at weeks 6 and 9. Final immune sera were collected at week 12 and purified using a two-step affinity purification with unmodified peptides, followed by modified peptides coupled to beads to obtain GG peptide–specific antibodies.

### Laser scanning confocal microscopy of fixed specimens

Cells were routinely fixed with 4% formaldehyde in PBS and washed once with PBS before storage at 4°C for further processing. To label intracellular compartments, cells were permeabilized and blocked (PBS, 0.1% Triton X-100, and 3% BSA) for 30 min, followed by an overnight incubation with primary antibodies diluted in 1% BSA-PBS at 4°C. On the next day, cells were incubated with secondary antibody diluted in 1% BSA-PBS for 1 hour at RT, before mounting on microscope slides using ProLong Glass Antifade Mountant containing NucBlue (Thermo Fisher Scientific). Relevant primary and secondary antibodies are shown in tables S4 and S5. Cells were imaged with confocal fluorescence microscopy using a Zeiss LSM 880 microscope fitted with a Zeiss 63× numerical aperture 1.4 objective operated in single-photon mode, with standard filter sets verified for lack of detectable cross-channel bleed through and standard (one airy disk) pinhole. Colocalization was quantified by Pearson’s coefficient with Costes threshold using the JaCOP plugin.

### Time-lapse imaging of α-synuclein dynamics

Cells were plated on a poly-l-lysine coated ibidi μ-Dish 35 mm, high Grid-500. Before imaging, media were replaced with phenol red free DMEM (Gibco) supplemented with NEAA and FBS. Time-lapse imaging was performed on cells 48 hours after transfection using Zeiss Cell Observer Spinning Disk Confocal. To study intraluminal localization of α-synuclein, HEK293T cells were transiently transfected with mRFP-tagged Rab5-Q79L and ecliptic GFP variant fused to the C terminus of α-synuclein. On the day of the experiment, cells were treated with 500 μM chloroquine, and images were acquired for 5 min with each frame taken every 5 s. Cross sections showing chloroquine-induced intraluminal fluorescence were processed using ZEN Blue software (Zeiss). Raw data were exported to TIFF (16-bit) format, and mean fluorescence intensity of individual endosomes before and after chloroquine treatment was measured using the ImageJ (Fiji) software. For data processing, selections were drawn around each endosome and the mean fluorescence values were background-corrected. Background-subtracted fluorescence intensities obtained from the first five frames of the image sequence were averaged and identified as the “minimum fluorescence value.” The maximal average fluorescence value was determined from the last five frames of the acquired image sequence. The fold increase for each endosome was determined from the average maximum fluorescence value divided by the average minimum fluorescence value.

### Staining of human brain sections

Brain tissue from three patients and one healthy control was obtained from the brain bank of the John Radcliffe Hospital in Oxford (table S6). Tissue was formalin-fixed, paraffin-embedded, and serially sectioned at 7 μm with a sliding microtome. Staining was performed using the Histostain-Plus detection kit (Life Technologies, Paisley, UK) and the HISTAR detection kit STAR3000C (AbD Serotec, Oxford, UK). Sections were dewaxed and rehydrated, and antigen unmasking was achieved by autoclaving. For immunofluorescent staining, to minimize tissue autofluorescence, an additional quenching step was performed by incubating the sections with 0.1% Sudan black for 5 min, followed by four 5-min washes in tris-buffered saline containing 0.05% Triton X-100 (TBS-T). Endogenous peroxidase activity was quenched in 3% (v/v) hydrogen peroxide, followed by incubation with the primary antibody in TBS-T at 4°C overnight. Washes were performed in TBS-T and PBS. The color reaction was developed with diaminobenzidine, and counterstaining was made in hematoxylin and eosin. Following washes, dehydration and Histo-Clear sections were mounted with glass coverslips. The slides were then scanned and visualized using the Aperio ScanScope AT Digital Pathology Slide Scanner.

### Statistical analysis

All statistical analyses were performed using GraphPad Prism 8 (GraphPad Software Inc., La Jolla, CA USA). All data were examined for normality, and statistical tests were chosen accordingly. For normally distributed data, one-way analysis of variance (ANOVA) was used. For non-normally distributed data, the Kruskal-Wallis test was used. Two-way ANOVA was used to determine significant effects of ubiquitination or degradation over a time course. Two-sided unpaired Student’s *t* test was used where indicated when two conditions were compared. The null hypothesis was rejected at a significance level of *P* = 0.05.

### Ethical approval

Ethical approval for the use of human tissue was obtained from the Oxford C REC Ethics Committee (no. 15/SC/0639).

## Notes

View/request a protocol for this paper from *Bio-protocol*.
