# Supplementary material for: Monitoring α-synuclein ubiquitination dynamics reveals key endosomal effectors mediating its trafficking and degradation
Source: Sci Adv. 2023 Jun 14;9(24):eadd8910. doi: 10.1126/sciadv.add8910 (PMC10266730; doi:10.1126/sciadv.add8910)
Supplement: Supplementary file 1 — Tables S1 to S6 Figs. S1 to S7 Legends for movies S1 to S11 [file sciadv.add8910_sm.pdf]

Science Advances
AAAS

Supplementary Materials for

Monitoring α-synuclein ubiquitination dynamics reveals key endosomal effectors mediating its trafficking and degradation

Dmitry Zenko *et al.*

Corresponding author: George K. Tofaris, george.tofaris@ndcn.ox.ac.uk

Sci. Adv. **9**, eadd8910 (2023)
DOI: 10.1126/sciadv.add8910

The PDF file includes:

Tables S1 to S6
Figs. S1 to S7
Legends for movies S1 to S11

Other Supplementary Material for this manuscript includes the following:

Movies S1 to S11

Supplementary Table 1: GO enrichment analysis for top protein hits interacting with ubiquitinated α-synuclein

<table>
<tr><th></th><th>GO_term_ID</th><th>GO_term_def</th><th>P</th><th>P_FDR_adj</th><th>NofGen</th><th>Genes</th></tr>
<tr><td>1</td><td>GO:0036019</td><td>endo-lysosome</td><td>0E+00</td><td>1.23E-08</td><td>7</td><td>AP2B1|AP2M1|AP2S1|CLTA|CLTC|CTSB|LGMN</td></tr>
<tr><td>2</td><td>GO:0030136</td><td>clathrin-coated vesicle</td><td>7.6E-05</td><td>3.36E-03</td><td>7</td><td>AP2B1|AP2M1|AP2S1|CLTA|CLTC|RAB5A|SNX9</td></tr>
<tr><td>3</td><td>GO:0005770</td><td>late endosome</td><td>5.22E-04</td><td>1.46E-02</td><td>7</td><td>LAMP2|LGMN|MAP2K1|MAP2K2|MAPK1|MAPK3|VPS4A</td></tr>
<tr><td>4</td><td>GO:0030135</td><td>coated vesicle</td><td>1.98E-04</td><td>6.78E-03</td><td>8</td><td>AP2B1|AP2M1|AP2S1|CLTA|CLTC|RAB5A|SEC31A|SNX9</td></tr>
<tr><td>5</td><td>GO:0005769</td><td>early endosome</td><td>1.46E-04</td><td>5.34E-03</td><td>9</td><td>MAP2K1|MAP2K2|MAPK1|MAPK3|MMGT1|RAB5A|SNX12|VPS4A|WASF2</td></tr>
<tr><td>6</td><td>GO:0010008</td><td>endosome membrane</td><td>6.19E-05</td><td>3.17E-03</td><td>11</td><td>AP2B1|AP2M1|AP2S1|CLTA|CLTC|LAMP2|MMGT1|RAB5A|SCAMP2|VPS4A|WDR44</td></tr>
<tr><td>7</td><td>GO:0044437</td><td>vacuolar part</td><td>6.33E-05</td><td>3.17E-03</td><td>12</td><td>AP2B1|AP2M1|AP2S1|CLTA|CLTC|CTSB|LAMP2|LGMN|MAPK1|PRCP|RHEB|VPS4A</td></tr>
<tr><td>8</td><td>GO:0030659</td><td>cytoplasmic vesicle membrane</td><td>9.88E-04</td><td>2.46E-02</td><td>12</td><td>ANXA7|AP2B1|AP2M1|AP2S1|CLTA|CLTC|LAMP2|MYO1C|PRCP|RAB5A|SEC31A|SNX9</td></tr>
<tr><td>9</td><td>GO:0005764</td><td>lysosome</td><td>8.51E-05</td><td>3.36E-03</td><td>13</td><td>AP2B1|AP2M1|AP2S1|CLTA|CLTC|CTSB|LAMP2|LGMN|MAPK1|PRCP|RHEB|TRIP10|VPS4A</td></tr>
<tr><td>10</td><td>GO:0005773</td><td>vacuole</td><td>3.27E-04</td><td>9.66E-03</td><td>13</td><td>AP2B1|AP2M1|AP2S1|CLTA|CLTC|CTSB|LAMP2|LGMN|MAPK1|PRCP|RHEB|TRIP10|VPS4A</td></tr>
<tr><td>11</td><td>GO:0005768</td><td>endosome</td><td>4.32E-07</td><td>8.5E-05</td><td>19</td><td>AP2B1|AP2M1|AP2S1|CLTA|CLTC|CTSB|LAMP2|LGMN|MAP2K1|MAP2K2|MAPK1|MAPK3|
MMGT1|RAB5A|SCAMP2|SNX12|VPS4A|WASF2|WDR44</td></tr>
<tr><td>12</td><td>GO:0097708</td><td>intracellular vesicle</td><td>3.25E-05</td><td>2.23E-03</td><td>28</td><td>ANXA7|AP2B1|AP2M1|AP2S1|ATP1B3|CLTA|CLTC|CRCP|CTSB|GNA13|LAMP2|LGMN|MAP2K1|
MAP2K2|MAPK1|MAPK14|MAPK3|MMGT1|MYO1C|PRCP|RAB5A|SCAMP2|SEC31A|SNX12|SNX9|
VPS4A|WASF2|WDR44</td></tr>
<tr><td>13</td><td>GO:0012505</td><td>endomembrane system</td><td>1.69E-03</td><td>3.85E-02</td><td>38</td><td>ABI1|ANXA7|AP2B1|AP2M1|AP2S1|APMAP|CLCC1|CLTA|CLTC|CRCP|CTSB|DHRS7B|FKBP7|IKBIP|
LAMP2|LGMN|MAP2K1|MAP2K2|MAPK1|MAPK14|MAPK3|MMGT1|MYO1C|NUP133|PLOD2|PRCP|
RAB5A|RHEB|SACM1L|SCAMP2|SEC31A|SNX12|SNX9|TRIP10|VPS4A|WASF2|WDR44|XPOT</td></tr>
<tr><td>14</td><td>GO:0031982</td><td>vesicle</td><td>2.62E-05</td><td>1.97E-03</td><td>39</td><td>ABHD14B|ABI1|ANXA7|AP2B1|AP2M1|AP2S1|ATP1B3|CLTA|CLTC|CRCP|CTSB|DPP3|GNA13|IDE|
LAMP2|LGMN|MAP2K1|MAP2K2|MAPK1|MAPK14|MAPK3|MMGT1|MYO1C|PLOD2|PRCP|RAB5A|
RDX|RHEB|SCAMP2|SEC31A|SLC7A5|SNX12|SNX9|TRIP10|UGP2|VPS13C|VPS4A|WASF2|WDR44</td></tr>
<tr><td>15</td><td>GO:0031974</td><td>membrane-enclosed lumen</td><td>1.33E-05</td><td>1.14E-03</td><td>49</td><td>ABHD14B|AQR|CPSF3|CRCP|CTSB|DDI2|DNMT1|DPP3|ECI2|FKBP7|GEMIN7|GSTZ1|HNRNPUL1|
IDE|ILKAP|LAMP2|LGMN|MAPK1|MAPK14|MAPK3|MDN1|MRPL15|MTHFD1L|MTHFD2|MYO1C|NDUFB6|
NTMT1|OPA1|ORC3|PAWR|PPME1|PRPF31|PTPN11|REXO2|RNASEH2B|RNF20|RPP30|RPRD2|SUPT6H|
TARS2|THOC1|THOP1|TIMELESS|TRIP10|TSFM|UBLCP1|XPOT|ZC3H18|ZWINT</td></tr>
<tr><td>16</td><td>GO:0070013</td><td>intracellular organelle lumen</td><td>1.33E-05</td><td>1.14E-03</td><td>49</td><td>ABHD14B|AQR|CPSF3|CRCP|CTSB|DDI2|DNMT1|DPP3|ECI2|FKBP7|GEMIN7|GSTZ1|HNRNPUL1|IDE|
ILKAP|LAMP2|LGMN|MAPK1|MAPK14|MAPK3|MDN1|MRPL15|MTHFD1L|MTHFD2|MYO1C|NDUFB6|
NTMT1|OPA1|ORC3|PAWR|PPME1|PRPF31|PTPN11|REXO2|RNASEH2B|RNF20|RPP30|RPRD2|SUPT6H|
TARS2|THOC1|THOP1|TIMELESS|TRIP10|TSFM|UBLCP1|XPOT|ZC3H18|ZWINT</td></tr>
<tr><td>17</td><td>GO:0044422</td><td>organelle</td><td>8.59E-07</td><td>1.29E-04</td><td>77</td><td>ABHD14B|ANXA7|AP2B1|AP2M1|AP2S1|AQR|CLTA|CLTC|CPSF3|CRCP|CTSB|DDI2|DHRS7B|DNMT1|
DPP3|ECI2|FKBP7|GEMIN7|GSTZ1|HNRNPUL1|IDE|IKBIP|ILKAP|L2HGDH|LAMP2|LGMN|MAP2K1|MAP2K2|
MAPK1|MAPK14|MAPK3|MDN1|MMGT1|MRPL15|MTHFD1L|MTHFD2|MYO1C|NDUFA12|NDUFB6|NDUFV1|
NTMT1|NUP133|OPA1|ORC3|PAWR|PLOD2|PPME1|PRCP|PRPF31|PTPN11|RAB5A|RDX|REXO2|RHEB|
RNASEH2B|RNF20|RPP30|RPRD2|SACM1L|SCAMP2|SEC31A|SNX9|SUPT6H|TACC3|TARS2|THOC1|
THOP1|TIMELESS|TRIP10|TSFM|UBLCP1|VPS13C|VPS4A|WDR44|XPOT|ZC3H18|ZWINT</td></tr>
</table>

Supplementary Table 2: siRNA sequences (Dharmacon, USA)

Gene Symbol	Sequence (5’-3’)	Catalogue No
Non-Target	UGGUUUACAUGUCGACUAA, UGGUUUACAUGUUGUGUGA<br>UGGUUUACAUGUUUUCUGA, UGGUUUACAUGUUUUCCUA	D-001810-10-05
ATG7	GAUCUAAAUCUCAAACUGA<br>GCCCACAGAUGGAGUAGCA<br>GCCAGAGGAUUCAACAUGA<br>CCAACACACUCGAGUCUUU	J-020112-05<br>J-020112-06<br>J-020112-07<br>J-020112-08
NBR1	GAGAACAAGUGGUUAACGA<br>CCACAUGACAGUCCUUUAA<br>GAACGUAUACUUCCCAUUG<br>AGAAGCCACUUGCACAUUA	L-010522-01<br>L-010522-02<br>L-010522-03<br>L-010522-04
HSC70	CGUAAUACCACCAUUCCUA<br>GAUAACAACCUGCUUGGCA<br>GACCUUCACUACCUAUUCU<br>UGAAACUGCUGGUGGAGUC	J-017609-06<br>J-017609-07<br>J-017609-08<br>J-017609-09
VPS13C	ACUAGAAGUUGGCGAGAUU<br>UGUCCAAAGUAGACGGCAA<br>ACUUAAGGUAGAAGCGAAA<br>AGAAAGAGCUAUCCCGAAU	J-020641-18<br>J-020641-19<br>J-020641-20<br>J-020641-21
CHMP3	GGGCAAAGCACCCAGUAAA<br>GAUCAUAGAGGAGAUGUUA<br>CAAGCUGUAUGCAUCCAAA<br>GAAAUGGAAAUUGACAGAA	J-004696-05<br>J-004696-06<br>J-004696-07<br>J-004696-08
Non-Target Accell	UGGUUUACAUGUUGUGUGA, UGGUUUACAUGUCGACUAA<br>UGGUUUACAUGUUUUCCUA, UGGUUUACAUGUUUUCUGA	D-001910-10-05
NBR1 Accell	GUAUCAUAGUGGAUCCUUU<br>GUCUUGGGAUCAGAUAUGA<br>GUAAAAGUUUCAUUUGAUC<br>GAUUCUUCCUUGAAAUUUG	A-085910-13<br>A-085910-14<br>A-085910-15<br>A-085910-16
Tsg101 Accell	CCGUUUAGAUCAAGAAGUA<br>CUCCCAAUCCCAGUGGUUA<br>CGUGAAACUGUCAAUGUUA<br>CAUUCAAGUGUAAAAUAUU	A-003549-14<br>A-003549-15<br>A-003549-16<br>A-003549-17

Supplementary Table 3: shRNA sequences (Thermo Scientific, USA)

Gene Symbol	Full Hairpin Sequence	Sense Sequence
NEDD4 sh1	TGCTGTTGACAGTGAGCGACTGGCTTTAGAGTTCACACAATAGTGAAGCC<br>ACAGATGTATTGTGTGAACTCTAAAGCCAGGTGCCTACTGCCTCGGA	TGGCTTTAGAGTTC<br>ACACA
NEDD4 sh2	TGCTGTTGACAGTGAGCGAATGGAAGAATCTTCTACATAATAGTGAAGCC<br>ACAGATGTATTATGTAGAAGATTCTTCCATCTGCCTACTGCCTCGGA	TGGAAGAATCTTCTA<br>CATA
SKP1 sh1	TGCTGTTGACAGTGAGCGCGCAGCAGCAAGTCAATTGTATTAGTGAAGCC<br>ACAGATGTAATACAATTGACTTGCTGCTGCATGCCTACTGCCTCGGA	CAGCAGCAAGTCAA<br>TTGTA
SKP1 sh2	TGCTGTTGACAGTGAGCGACCAAACAATCTGTGACTATTATAGTGAAGCC<br>ACAGATGTATAATAGTCACAGATTGTTTGGCTGCCTACTGCCTCGGA	CAAACAATCTGTGA<br>CTATT
CHIP sh1	TGCTGTTGACAGTGAGCGACCAGCTGGAGATGGAGAGCTATAGTGAAGC<br>CACAGATGTATAGCTCTCCATCTCCAGCTGGCTGCCTACTGCCTCGGA	CAGCTGGAGATGGA<br>GAGCT
CHIP sh2	TGCTGTTGACAGTGAGCGCTGACGCATTCATCTCTGAGAATAGTGAAGCC<br>ACAGATGTATTCTCAGAGATGAATGCGTCAATGCCTACTGCCTCGGA	GACGCATTCATCTC<br>TGAGA
HRS sh1	TGCTGTTGACAGTGAGCGACCCCAGAATGGTACAGCGATATAGTGAAGC<br>CACAGATGTATATCGCTGTACCATTCTGGGGCTGCCTACTGCCTCGGA	CCCAGAATGGTACA<br>GCGAT
HRS sh2	TGCTGTTGACAGTGAGCGCCCGGAACGAGCCCAAGTACAATAGTGAAGC<br>CACAGATGTATTGTACTTGGGCTCGTTCCGGATGCCTACTGCCTCGGA	CGGAACGAGCCCAA<br>GTACA
STAM sh1	TGCTGTTGACAGTGAGCGATTCGATCAGGATGTTGAGAAATAGTGAAGCC<br>ACAGATGTATTTCTCAACATCCTGATCGAAGTGCCTACTGCCTCGGA	TCGATCAGGATGTT<br>GAGAA
STAM sh2	TGCTGTTGACAGTGAGCGAGGTCATCCTAAAGTATGTGAATAGTGAAGCC<br>ACAGATGTATTCACATACTTTAGGATGACCCTGCCTACTGCCTCGGA	GTCATCCTAAAGTAT<br>GTGA

Gene Symbol	Full Hairpin Sequence	Sense Sequence
p62 sh1	TGCTGTTGACAGTGAGCGAACCCATCTGTCTTCAAAAGAATAGTGAAGCC ACAGATGTATTCTTTTGAAGACAGATGGGTCTGCCTACTGCCTCGGA	CCCATCTGTCTTCAA AAGA
p62 sh2	TGCTGTTGACAGTGAGCGCTCCGAGTGTGAATTTCCTGAATAGTGAAGCC ACAGATGTATTCAGGAAATTCACACTCGGATTGCCTACTGCCTCGGA	CCGAGTGTGAATTT CCTGA
OPTN sh1	TGCTGTTGACAGTGAGCGCCAGCGAAGCTGAGCTAATGAATAGTGAAGC CACAGATGTATTCATTAGCTCAGCTTCGCTGATGCCTACTGCCTCGGA	AGCGAAGCTGAGCT AATGA
OPTN sh2	TGCTGTTGACAGTGAGCGCAAGCATGCTATCAGAAATCAATAGTGAAGCC ACAGATGTATTGATTTCTGATAGCATGCTTTTGCCTACTGCCTCGGA	AGCATGCTATCAGA AATCA
NDP52	TGCTGTTGACAGTGAGCGCCAGGGAGATCAAGATAAGACATAGTGAAGC CACAGATGTATGTCTTATCTTGATCTCCCTGATGCCTACTGCCTCGGA	AGGGAGATCAAGAT AAGAC
NDP52	TGCTGTTGACAGTGAGCGAAAGCTTGTTCAGGGAGATCAATAGTGAAGCC ACAGATGTATTGATCTCCCTGAACAAGCTTCTGCCTACTGCCTCGGA	AGCTTGTTCAGGGA GATCA
TAX1BP1	TGCTGTTGACAGTGAGCGCAAGAAGAACTGTTAAAGTTAATAGTGAAGCC ACAGATGTATTAACTTTAACAGTTCTTCTTTTGCCTACTGCCTCGGA	AGAAGAACTGTTAA AGTTA
TAX1BP1	TGCTGTTGACAGTGAGCGACTGGAATGTCATTACACCTTATAGTGAAGCC ACAGATGTATAAGGTGTAATGACATTCCAGGTGCCTACTGCCTCGGA	TGGAATGTCATTACA CCTT
TSG101	TGCTGTTGACAGTGAGCGCACCCGTTTAGATCAAGAAGTATAGTGAAGCC ACAGATGTATACTTCTTGATCTAAACGGGTATGCCTACTGCCTCGGA	CCCGTTTAGATCAA GAAGT
NBR1	TGCTGTTGACAGTGAGCGACTGGAGACGTTCAGAGAACAATAGTGAAGC CACAGATGTATTGTTCTCTGAACGTCTCCAGGTGCCTACTGCCTCGGA	TGGAGACGTTCAGA GAACA
NBR1	TGCTGTTGACAGTGAGCGAAAGGATTGTTGGTGTCCGCTATAGTGAAGCC ACAGATGTATAGCGGACACCAACAATCCTTCTGCCTACTGCCTCGGA	AGGATTGTTGGTGT CCGCT

Supplementary Table 4: Primary antibodies

Antibody	Supplier/Catalogue number	Host	WB	ICC
**Anti-α-Synuclein Clone 42/(RUO)**	BD Transduction Laboratories™/610786	Mouse	1:1000	1:1000
**Anti-pSyn EP1536Y antibody**	Abcam/ab51253	Rabbit	1:1000	-
**Anti-α-Synuclein Clone MJFR1**	Abcam/ ab138501	Rabbit	1:1000	-
**anti-UbSyn^{3K}**	Made in house	Rabbit	1:500	1:100-1:500
**Ubiquitin (P4D1)**	Cell signalling technologies (CST)/3936	Mouse	1:2000	1:500-1:1000
**Anti-GFP, N-terminal antibody**	Sigma Aldrich/G1544-100UG	G1544-100UG	1:2000	1:2000
**Monoclonal Anti-β-Actin Clone AC-15**	Sigma Aldrich/A5441	Mouse	1:10 000	-
**Neuron-specific beta-III Tubulin**	Bio-Techne Ltd./MAB1195-SP	Mouse	1:5000	1:1000
**Anti-MAP2**	Abcam/ab5392	Chicken	-	1:1500
**Anti-HRS (D7T5N) mAb**	Cell signalling technologies (CST)/15087	Rabbit		1:1000
**Anti-NBR1 [5C3]**	Abcam/ab55474	Mouse	1:500	1:500
**Anti-LC3B [GT3612]**	Abcam/ab243506	Mouse	1:3000	1:1000
**Anti-SQSTM1 /p62 [2C11]**	Abcam/ab56416	Mouse	-	1:500
**K48-linkage Specific Polyubiquitin (D9D5)**	Cell signalling technologies (CST)//8081	Rabbit	1:1000	1:500
**K63-linkage Specific Polyubiquitin (D7A11)**	Cell signalling technologies (CST)/12930	Rabbit	1:1000	1:500
**Rab5 (C8B1) mAb**	Cell signalling technologies (CST)/3547	Rabbit	-	1:200

Antibody	Supplier/Catalogue number	Host	WB	ICC
**Rab7 (D95F2) XP® mAb**	Cell signalling technologies (CST)/9367	Rabbit	1:1000	1:200
**LAMP1 (D4O1S) mAb**	Cell signalling technologies (CST)/15665	Mouse	1:1000	1:200
**Anti-GAPDH**	Abcam/ab9485	Rabbit	1:2000	-
**Anti-tyrosine hydroxylase clone [illegible]**	Millipore #MAB318	Mouse	-	1:250
**Anti-βIII tubulin/TUJ1**	BioLegend #802001	Rabbit	-	1:1500
**Anti-SQSTM1 / p62 antibody [2C11]**	Abcam/ab56416	Mouse	1:1000	-
**Anti-NDP52 [GT422]**	GeneTex/GTX630396	Mouse	1:1000	-
**Anti-Nedd4**	Sigma-Aldrich/07-049	Rabbit	1:1000	-
**Anti-OPTN**	Sino Biological/14478-MM08	Mouse	1:1000	-
**Anti-STAM Antibody (B-2)**	Santa Cruz/sc-133093	Mouse	1:500	-
**Anti- LC3B (E5Q2K)**	Cell signaling technologies (CST)/ #83506	Mouse	-	1:200
**Anti-Skp1 p19**	Santa Cruz/sc-5281	Mouse	1:500	-
**Anti-CHIP (C-10)**	Santa Cruz/sc-1333083	Mouse	1:500	-
**Anti-Cas9 (7A9-3A3)**	Cell signaling technologies (CST)/ #14697	Mouse	1:1000	-
**Anti-TSG101 (4A10)**	GeneTex/GTX70255	Mouse	1:1000	1:1000
**Anti-Rab1A (D3X9S)**	Cell signaling technologies (CST)/13075	Raabit	-	1:500
**Anti-VPS13C**	Proteintech/28676-1-AP	Rabbit	1:1000	1:1000
**Anti-VPS24**	Proteintech/15472-1-AP	Rabbit	1:1000	-
**NBR1 (4BR)**	Santa Cruz/ sc-130380	Mouse	1:200	-
**Proteasome 20S alpha + beta**	Abcam/ ab22673	Rabbit	-	1:1000

Supplementary Table 5: Secondary antibodies

Antibody	Supplier/Catalogue number	Dilution
**Goat Anti-Mouse IgG H&L (HRP)**	Abcam/ab6789	1:5000
**Goat Anti-Rabbit IgG H&L (HRP)**	Abcam/ab6721	1:5000
**Goat Anti-rabbit IgG (H+L) (DyLight™ 800 4X PEG**	Cell signaling technologies/5151	1:10000
**Goat Anti-mouse IgG (H+L) (DyLight™ 680 Conjugate)**	Cell signaling technologies/5470	1:10000
**AlexaFluor® 488 Goat anti-Mouse IgG (H+L)**	Invitrogen/A11001	1:1000
**AlexaFluor® 488 Goat anti-Rabbit IgG (H+L)**	Invitrogen/A11008	1:1000
**AlexaFluor® 568 Goat anti-Mouse IgG (H+L)**	Invitrogen/ A11004	1:1000
**AlexaFluor® 568 Goat anti-Rabbit IgG (H+L)**	Invitrogen/ A11011	1:1000

Antibody	Supplier/Catalogue number	Dilution
**Goat Anti-Mouse IgG H&L (Alexa Fluor® 647)**	ab150115	1:1000
**Goat Anti-Rabbit IgG H&L (Alexa Fluor® 647)**	ab150079	1:1000
**Goat Anti-Rabbit IgG H&L (Alexa Fluor® 594)**	ab150080	1:1000
**Goat Anti-Mouse IgG H&L (Alexa Fluor® 594)**	ab150116	1:1000
**Goat Anti-Chicken IgY H&L (Alexa Fluor® 488)**	ab150169	1:1000
**Alexa Fluor® 647 Goat anti-Chicken IgY H&L**	Abcam/ab150171	1:1000

Supplementary Table 6: Parkinson's disease post-mortem brains

Case	Diagnosis	Sex	Age (years)	Brain (g)	PMI (hours)	CERAD	Braak PD	Braak AD
1	PD	m	92	1372	36	0	3	1
2	PD	m	92	1498	24	0	2	1
3	PD	m	71	1269	88	0	6	1

Supplementary Figures

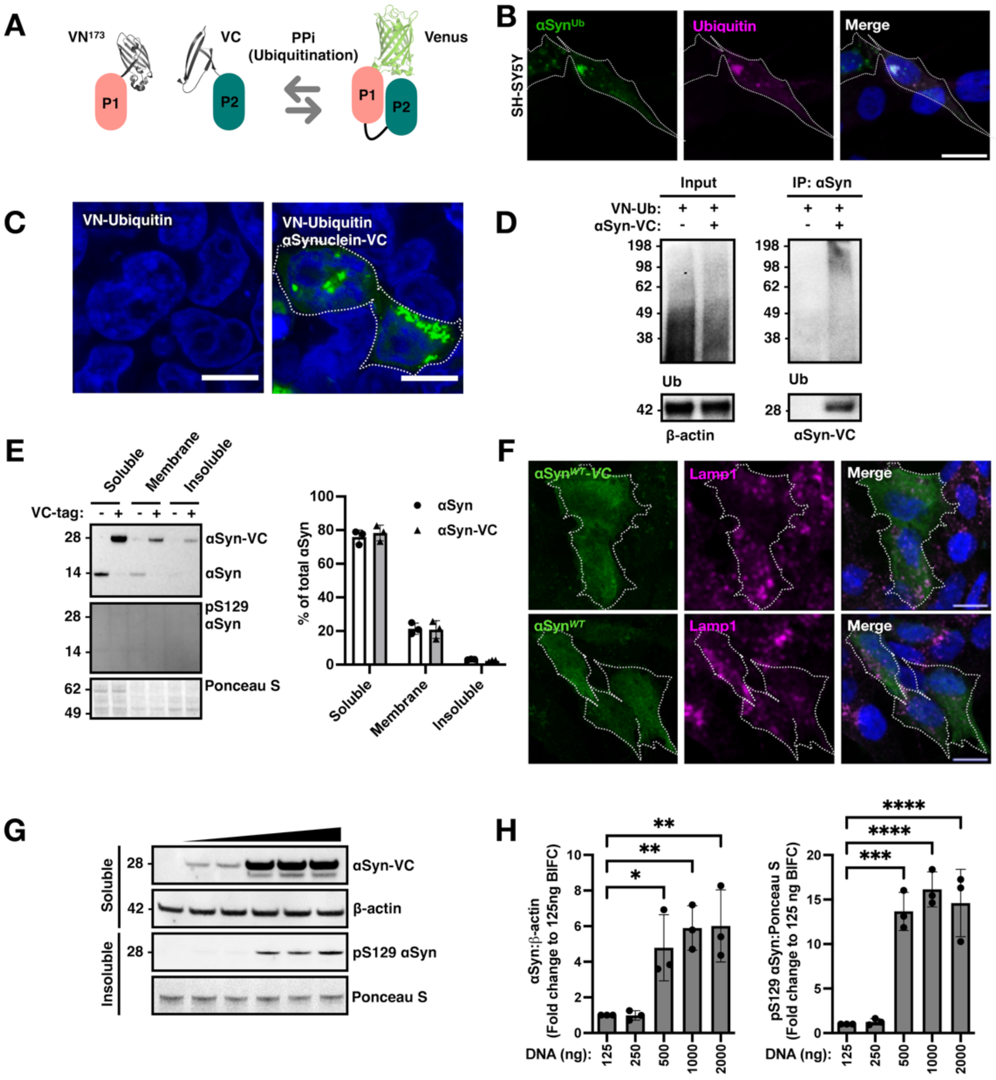

Fig. S1: Characterisation of the complementation assay. (A) Schematic illustrating the principle of bimolecular fluorescence complementation (BiFC). Venus fragment sequences were fused to the N-terminal of ubiquitin (VN173) and C-terminal of α-synuclein (VC155) and cloned into the pIRES2 bicistronic vector to drive the simultaneous expression of both fusion proteins under the same promoter. **(B)** Foci of BiFC signal form irrespective of the cell type used and were detected in SH-SY5Y cells shown here as well as HEK293 cells shown in Fig 1. **(C)** Puncta were detected when both α-synuclein-VC and VN-Ubiquitin were co-expressed. **(D)** Immunoprecipitation with the anti-α-synuclein antibody Syn1 revealed a ubiquitin positive smear (ubiquitinated α-synuclein) only when both VN-Ubiquitin and α-synuclein-VC were expressed in HEK293 cells. No smear was seen in the absence of α-synuclein-VC expression. **(E)** HEK293 cells expressing either α-synuclein-VC or untagged α-synuclein were lysed and fractionated into cytosolic, membrane or insoluble fractions. The relative distribution of these proteins across each fraction is the same and no pSer129 band was detected (n=3 independent experiments). **(F)** Expression of either α-synuclein-VC or untagged α-synuclein in SH-SY5Y knockout for endogenous α-synuclein shows that the VC tag *per se* does not increase association with lysosomes. **(G)** HEK293 cells expressing low and high levels of BiFC were lysed and fractionated into RIPA soluble and RIBA insoluble/SDS soluble fraction following 100,000g centrifugation and quantified in **(H)**. At low levels used throughout this study, α-synuclein-VC is soluble and not phosphorylated at Ser129. Higher expression causes aggregation (n=3 independent experiments).

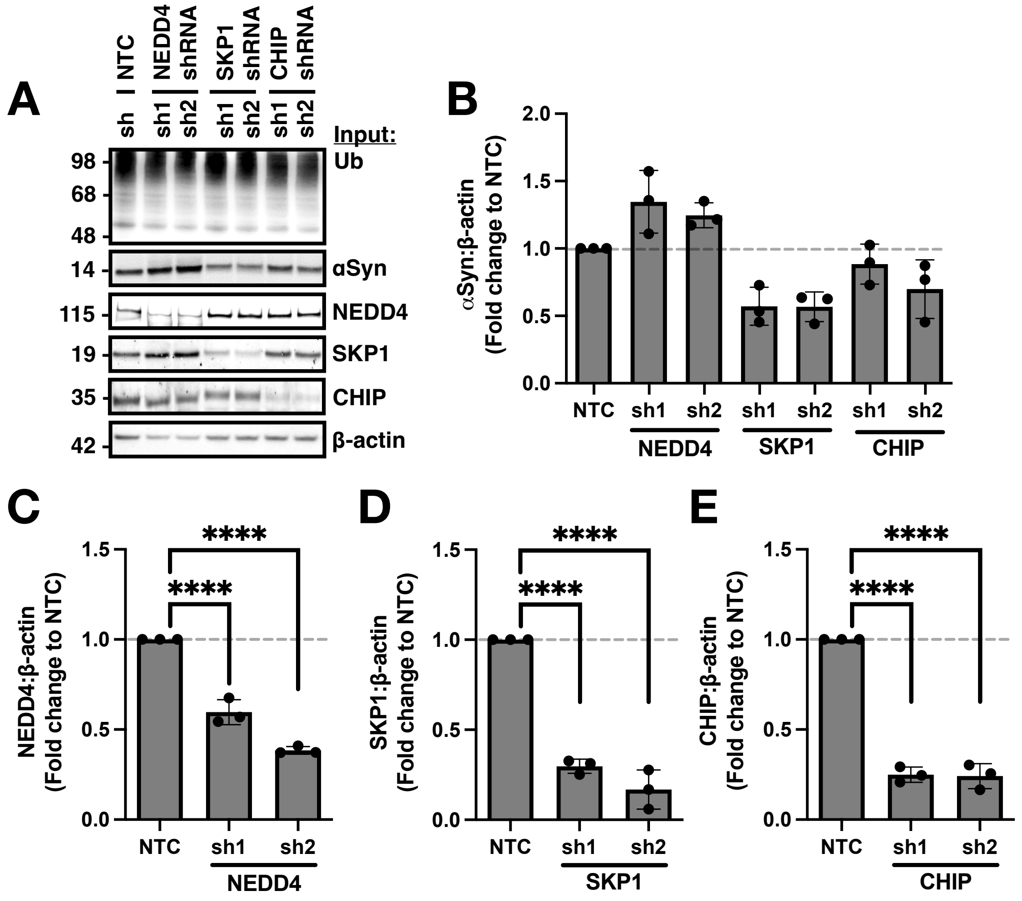

Fig S2: Knockdown of E3 ligases and their effect on α-synuclein levels. (A) Representative immunoblot showing two shRNAs per target that were used in triplicate to knock down the indicated E3 ligases. Note that the total levels of ubiquitination were unchanged under these conditions. **(B)** Levels of α-synuclein with each E3 ligase knockdown. Graphs showing the quantification of the knockdown of **(C)** NEDD4, **(D)** SKP1 and **(E)** CHIP (n=3 independent experiments for each shRNA).

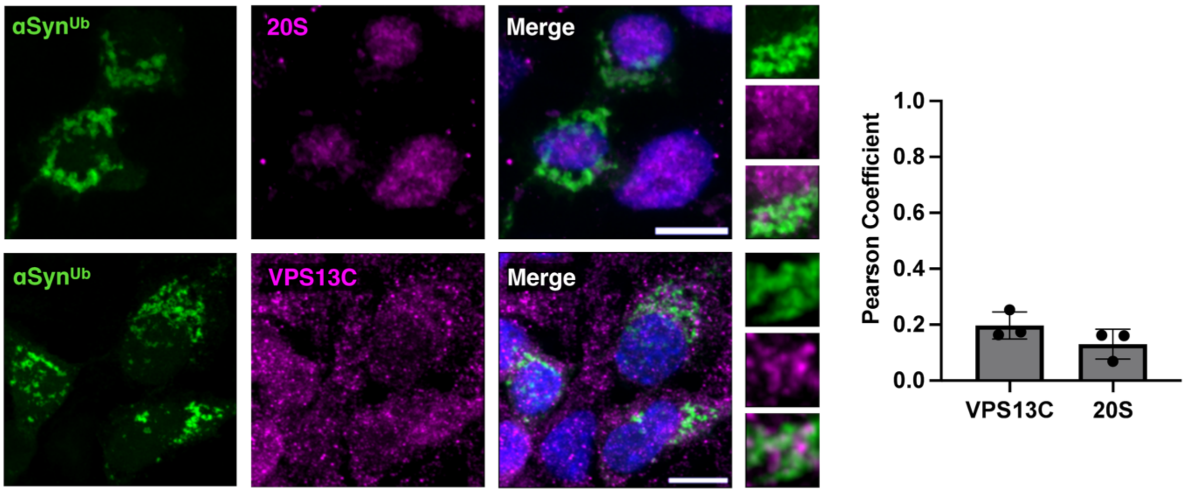

Fig. S3: Assessment of BiFC co-localisation with 20S proteasomes and VPS13C. Ubiquitinated α-synuclein (BiFC puncta) in HEK293 cells co-localised only minimally with the 20S proteasome or VPS13C as shown in the images and quantified in the graph based on n=3 independent experiments.

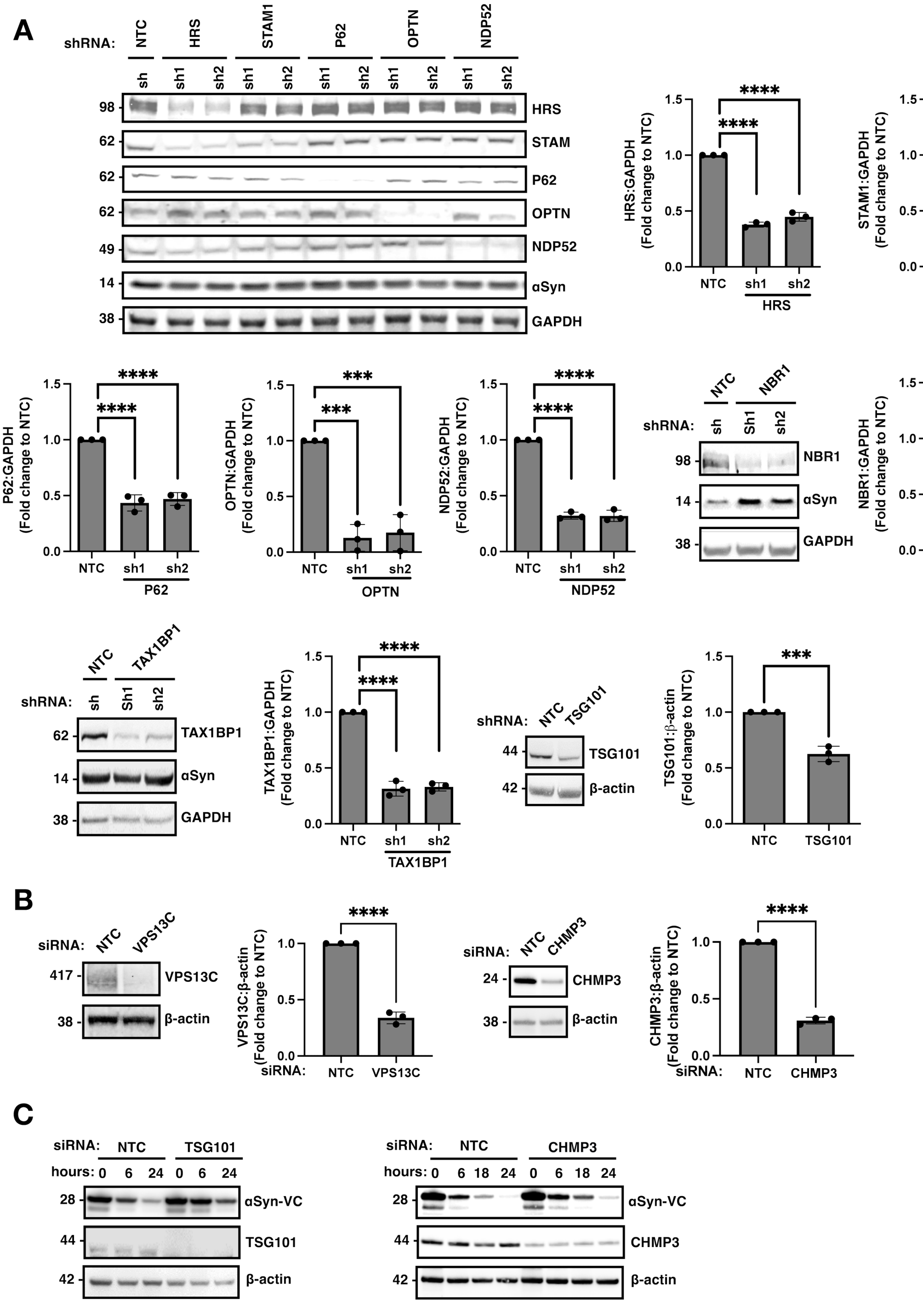

Fig. S4: Knockdown of relevant adaptors and their effect on α-synuclein. (A) Representative immunoblots confirming shRNA mediated knockdown of the protein adaptors. Representative immunoblots also show α-synuclein levels upon adaptor knockdown except for TSG101 knockdown for which α-synuclein levels are shown in Fig. 4L. Graphs show the quantification of the efficiency of each shRNA in triplicate for HRS, STAM1, p62, optineurin (OPTN) NDP52, NBR1, TAX1BP1 and TSG101. **(B)** Western blots and corresponding graphs showing the efficiency of siRNA-mediated VPS13C and CHMP3 knockdown. **(C)** Representative immunoblots showing cycloheximide chase experiments for α-synuclein levels at the indicated time points following knockdown of TSG101 or CHMP3 with corresponding quantifications in Fig. 4K and 4M respectively.

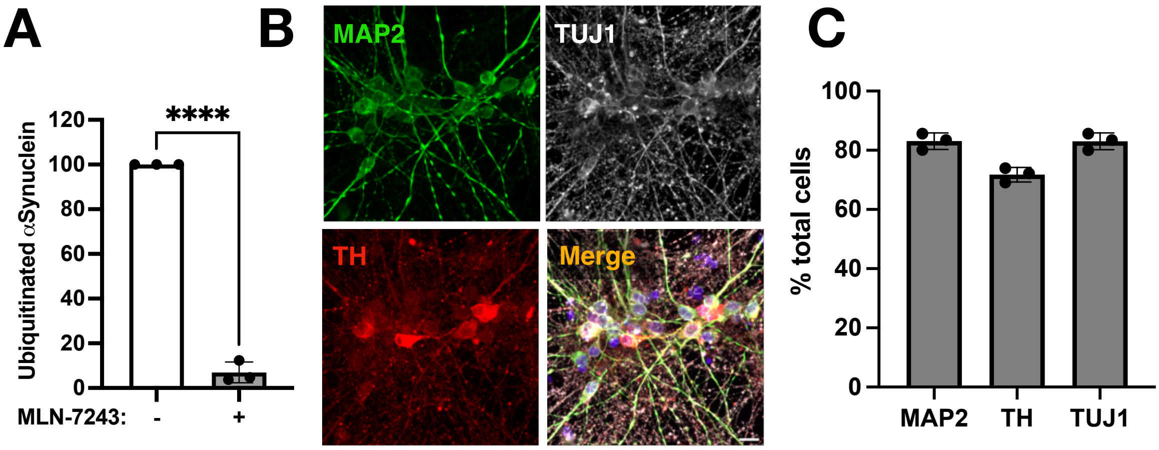

Fig. S5: Quantification of ubiquitinated α-synuclein and cell numbers in neuronal cultures. **A)** Quantification of the high MW smear (ubiquitinated α-synuclein) shown in Fig. 7C from three independent neuronal cultures following MLN-7243 treatment **(B)** Characterization of iPSC-derived dopaminergic neurons and **(C)** quantification of the percentage of TH-positive and MAP2-positive neurons to total cells based on DAPI (n=3 independent differentiations).

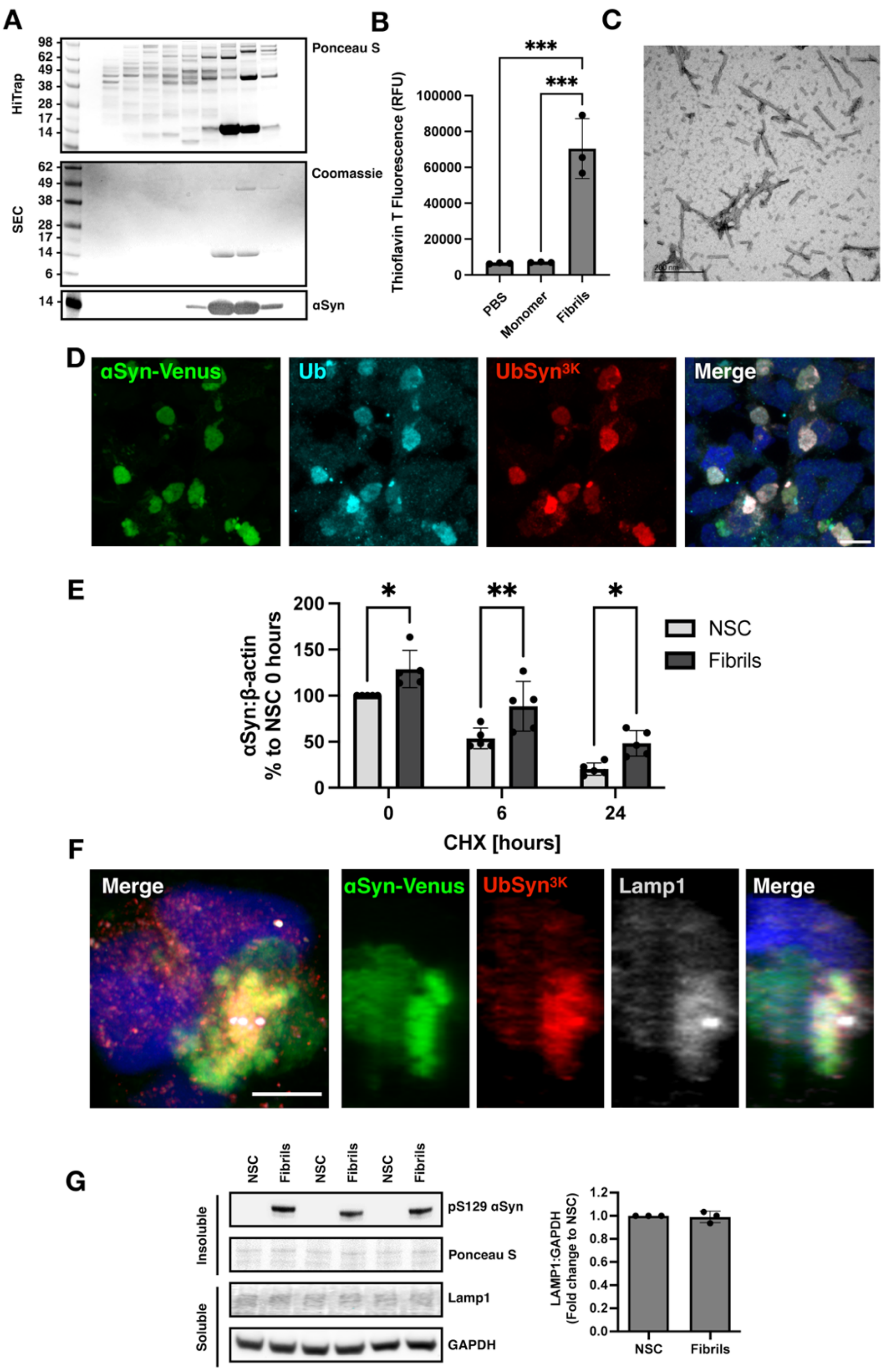

Fig. S6: Generation of fibrils and impact of fibril-induced aggregation on ubiquitinated α-synuclein. **(A)** Purification of recombinant α-synuclein using HiTrap followed by SEC and confirmation by immunoblotting with Syn1 antibody. Generation of seeding competent fibrils from monomeric α-synuclein was confirmed with **(B)** Thioflavin T fluorescence, **(C)** Transmission electron microscopy and **(D)** detection of seeding-induced anti-UbSyn^{3K} positive and ubiquitin-positive inclusions in HEK293 cells stably expressing α-synuclein-Venus. **(E)** Quantification of total α-synuclein-VC levels showing increased levels at each timepoint after seeded aggregation when compared to corresponding non-seeded control cells (n=5, representative immunoblot in Fig. 8C) **(F)** Confocal images of triple-labeled seeded HEK293 cells with orthogonal views demonstrate co-localisation between αSyn-Venus (green), UbSyn^{3K} (red), Lamp1 (white) **(G)** No change in Lamp1 levels was detected by immunoblotting after seeding.

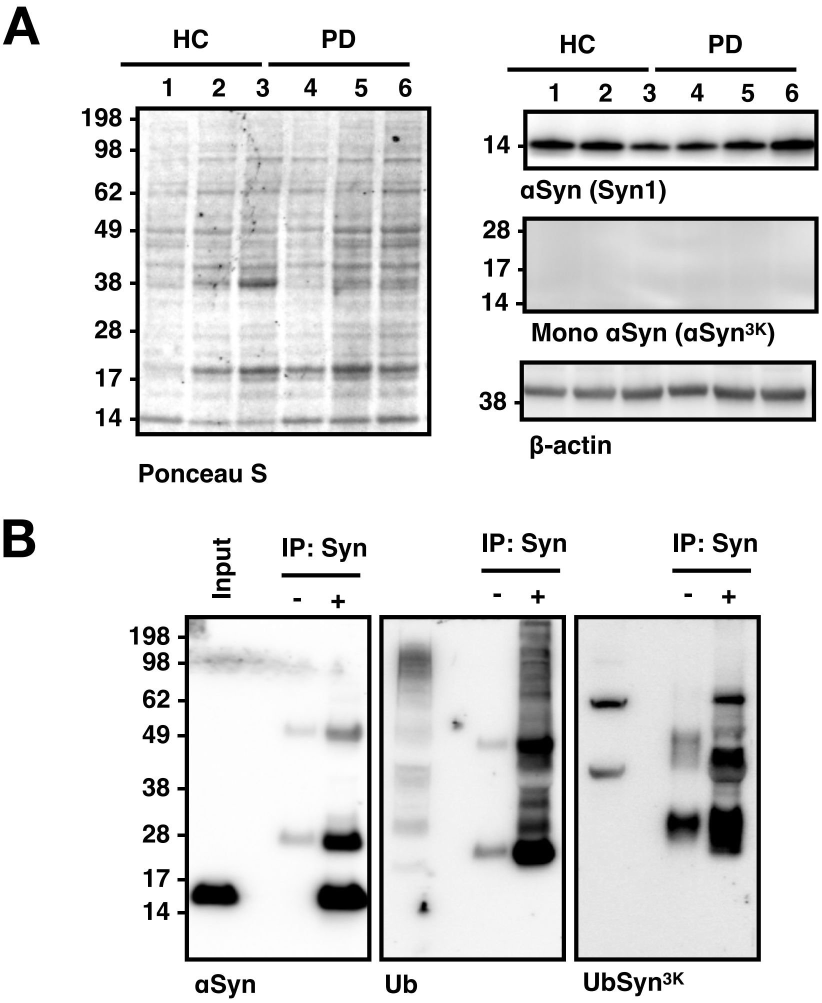

Fig. S7: Detection of ubiquitinated α-synuclein in human brain lysate. (A) Ponceau stain of human brain lysates and immunoblots with Syn1 (total α-synuclein), anti-UbSyn^{3K} antibody and actin. Note that the anti-UbSyn^{3K} antibody does not recognise monomeric (non-ubiquitinated) α-synuclein. **(B)** Immunoprecipitation with Syn1 antibody from human brain lysate followed by immunoblotting with Syn1, anti-Ubiquitin or anti-UbSyn^{3K}. Note that unlike Syn1, anti-UbSyn^{3K} recognises higher MW bands and a weak smear but not the monomer at 14 kDa.

Supplementary Videos

Video S1: Cells were treated with MLN7243 and subjected to time-lapse imaging for 2 hours with each frame taken every 10 seconds using Zeiss Cell Observer Spinning Disc Confocal. Representative video demonstrates a time-dependent decrease in BiFC signal in response to MLN7243 demonstrating that ubiquitination of α-synuclein is fast and dynamic.

Video S2: Cells expressing wild-type VN-Ubiquitin and α-Synuclein-VC constructs were incubated with 1μM of LysoTracker® DND-Red (LTR) and assessed with time-lapse imaging for 2 hours with each frame taken every 10 seconds. Representative video demonstrates fusion of BiFC-positive vesicles with LTR-stained organelles which signify late endosomes or lysosomes.

Video S3: Cells were transiently-transfected with Rab5^{Q79L} that enlarges early endosomes and super-ecliptic GFP variant (pHluorin) fused to the C-terminus of wild-type α-synuclein. On the day of the experiment, cells were treated with 500µM chloroquine and images were acquired for 5 min with each frame taken every 5 seconds. Cross-sections showing endosomal membrane localisation and chloroquine-induced intraluminal fluorescence of wild-type α-synuclein were processed using the Zeiss Blue software.

Video S4: Cells were transiently-transfected with Rab5^{Q79L} that enlarges early endosomes and super-ecliptic GFP variant (pHluorin) fused to the C-terminus of 3KR α-synuclein (Lysine residues 45, 58 and 60 mutated to Arginine). On the day of the experiment, cells were treated with 500µM chloroquine and images were acquired for 5 min with each frame taken every 5 seconds. No membrane or intraluminal fluorescence was seen, demonstrating that the endosomal membrane localisation and involution of α-synuclein is ubiquitin-dependent and specifically requires ubiquitination at lysine residues at position 45, 58 and 60.

Video S5: Cells treated with siRNA to knockdown NBR1 were transiently-transfected with Rab5^{Q79L} that enlarges early endosomes and super-ecliptic GFP variant (pHluorin) fused to the C-terminus of wild-type α-synuclein. On the day of the experiment, cells were treated with 500µM chloroquine and images were acquired for 5 min with each frame taken every 5 seconds. Cross-sections showing lack of chloroquine-induced intraluminal fluorescence of wild-type α-synuclein in NBR1 knockdown cells as processed using the Zeiss Blue software.

Video S6: Cells treated with siRNA NT control were transiently-transfected with Rab5^{Q79L} that enlarges early endosomes and super-ecliptic GFP variant (pHluorin) fused to the C-terminus of wild-type α-synuclein. On the day of the experiment, cells were treated with 500µM chloroquine and images were acquired for 5 min with each frame taken every 5 seconds. Cross-sections showing endosomal membrane localisation and chloroquine-induced intraluminal fluorescence of wild-type α-synuclein as processed using the Zeiss Blue software.

Video S7: Cells treated with siRNA to knockdown NBR1 were transiently-transfected with BiFC and then incubated with 1μM of LysoTracker® DND-Red (LTR). Cells were assessed with time-lapse imaging for 2 hours with each frame taken every 10 seconds. Representative video demonstrates lack of fusion of BiFC-positive vesicles with LTR-stained organelles which signify late endosomes or lysosomes.

Video S8: Cells treated with siRNA for NT control were transiently-transfected with BiFC and then incubated with 1μM of LysoTracker® DND-Red (LTR). Cells were assessed with time-lapse imaging for 2 hours with each frame taken every 10 seconds. Representative video demonstrates fusion of BiFC-positive vesicles with LTR-stained organelles which signify late endosomes or lysosomes as also seen in Video S2.

Video S9: Cells treated with siRNA to knockdown Tsg101 were transiently-transfected with $Rab5^{Q79L}$ that enlarges early endosomes and super-ecliptic GFP variant (pHluorin) fused to the C-terminus of wild-type α-synuclein. On the day of the experiment, cells were treated with 500μM chloroquine and images were acquired for 5 min with each frame taken every 5 seconds. Cross-sections showing endosomal membrane localisation and chloroquine-induced intraluminal fluorescence of wild-type α-synuclein as processed using the Zeiss Blue software.

Video S10: Cells treated with siRNA to knockdown CHMP3/VPS24 were transiently-transfected with $Rab5^{Q79L}$ that enlarges early endosomes and super-ecliptic GFP variant (pHluorin) fused to the C-terminus of wild-type α-synuclein. On the day of the experiment, cells were treated with 500μM chloroquine and images were acquired for 5 min with each frame taken every 5 seconds. Cross-sections showing endosomal membrane localisation and chloroquine-induced intraluminal fluorescence of wild-type α-synuclein as processed using the Zeiss Blue software.

Video S11: Cells treated with siRNA to knockdown VPS13C were transiently-transfected with $Rab5^{Q79L}$ that enlarges early endosomes and super-ecliptic GFP variant (pHluorin) fused to the C-terminus of wild-type α-synuclein. On the day of the experiment, cells were treated with 500μM chloroquine and images were acquired for 5 min with each frame taken every 5 seconds. Cross-sections showing endosomal membrane localisation and chloroquine-induced intraluminal fluorescence of wild-type α-synuclein as processed using the Zeiss Blue software.
